# Assistance level quantification-based human-robot interaction space reshaping for rehabilitation training

**DOI:** 10.3389/fnbot.2023.1161007

**Published:** 2023-05-02

**Authors:** Xiangyun Li, Qi Lu, Peng Chen, Shan Gong, Xi Yu, Hongchen He, Kang Li

**Affiliations:** ^1^West China Biomedical Big Data Center, West China Hospital, Sichuan University, Chengdu, China; ^2^Med-X Center for Informatics, Sichuan University, Chengdu, China; ^3^Sichuan University-Pittsburgh Institute, Sichuan University, Chengdu, China; ^4^School of Mechanical Engineering, Southwest Jiaotong University, Chengdu, China; ^5^Department of Orthopedic Surgery and Orthopedic Research Institute, West China Hospital, Sichuan University, Chengdu, China; ^6^Department of Rehabilitation Medicine, West China Hospital, Sichuan University, Chengdu, China; ^7^School of Rehabilitation Sciences, West China School of Medicine, Sichuan University, Chengdu, China; ^8^Key Laboratory of Rehabilitation Medicine in Sichuan Province, West China Hospital, Sichuan University, Chengdu, China

**Keywords:** human-robot interaction, rehabilitation training, AAN, assistance level quantification, interaction space reshaping, EMG

## Abstract

Stroke has become a major disease that seriously threatens human health due to its high incidence and disability rates. Most patients undergo upper limb motor dysfunction after stroke, which significantly impairs the ability of stroke survivors in their activities of daily living (ADL). Robots provide an optional solution for stroke rehabilitation by attending therapy in the hospital and the community, however, the rehabilitation robot still has difficulty in providing needed assistance interactively like human clinicians in conventional therapy. For safe and rehabilitation training, a human-robot interaction space reshaping method was proposed based on the recovery states of patients. According to different recovery states, we designed seven experimental protocols suitable for distinguishing rehabilitation training sessions. To achieve assist-as-needed (AAN) control, a PSO-SVM classification model and an LSTM-KF regression model were introduced to recognize the motor ability of patients with electromyography (EMG) and kinematic data, and a region controller for interaction space shaping was studied. Ten groups of offline and online experiments and corresponding data processing were conducted, and the machine learning and AAN control results were presented, which ensured the effective and the safe upper limb rehabilitation training. To discuss the human-robot interaction in different training stages and sessions, we defined a quantified assistance level index that characterizes the rehabilitation needs by considering the engagement of the patients and had the potential to apply in clinical upper limb rehabilitation training.

## 1. Introduction

Stroke has become a major disease that seriously threatens human health due to its high incidence and disability rates. Most stroke patients are accompanied by upper limb motor dysfunction, which brings great pain to their minds and bodies (Benjamin et al., 2017). Loss of upper limb function significantly impairs the ability of stroke survivors in their activities of daily living (ADL). Currently, the shortage of physical therapists becomes a problem with the increasing number of stroke patients. The process of stroke recovery has its complexity which could be divided into different stages (Malouin et al., 1994), and the patients have respective safe movement spaces in each stage. Robots provide an optional solution for stroke rehabilitation by attending therapy in the hospital and the community, but the outcomes of rehabilitation robot for upper limbs only produces limited improvement compared to conventional therapy (Berning et al., 2021). The intensive and repetitive training from the robots has positive effects on the restoration of functional abilities of human limbs (Kapsalyamov et al., 2019), and the participation of the patients in movement execution plays a more important role in neuroplasticity (Mark et al., 2006). However, the excessive assistance of the robots might lead to excessive dependence of the patients on the robots, and most rehabilitation robots cannot directly sense the engagement of patients like human therapists by conversation and observation, thus it is difficult to ensure the effectiveness without providing appropriate assistance level for the control of robots in the training sessions.

Motor ability reflects the patient's intention and capacity to actively participate in movement (Wang et al., 2022). For stroke rehabilitation, the involvement of muscle activities in active movements is difficult to be discerned by the measurement of external force alone. Thus, Physiological signals are also needed to perceive the muscle activity state in rehabilitation, and it requires electromyography (EMG) to interpret the muscle co-activation (Nordin et al., 2014). EMG is widely used in clinical fields because it can describe the intention of movement even when no motion occurs, and produce less delay for the online system (Lo and Xie, 2012) or trigger the FES (Li et al., 2022). EMG signal could be converted into muscle forces and moments by considering the physiological and biomechanical characteristics of the participants (Nasr et al., 2021), which facilitates to decode of the intentions of different patients for the customized controller of the robots. It would bring a positive influence on the patients by characterizing their motor ability and rehabilitation needs to formulate the control strategy of the rehabilitation robots (Qian et al., 2019), and compliance control could be achieved by adjusting the needed assistance amount in permissible human-robot interaction space.

For the continuous recovery of motor ability during rehabilitation, it is necessary to make corresponding modifications in the control parameters of the rehabilitation robot by obtaining the variation of the patients' motor ability and motion intention through EMG, which provides an adaptable way to meet the needs from the patients in different training stages and sessions of rehabilitation. Thus, an increasing number of control strategies are introduced to the rehabilitation robots application. An exoskeleton controller based on adaptive technology is developed (Proietti et al., 2015), which can obtain the muscle activity of the participant through EMG and adjust the assistance level in passive assistive and active assistive modes. In addition, a review mentioned that the resistive haptic training strategies might have great potential to increase patients' attention and motor learning, however, the proposed taxonomy does not include the outcome metric in this work such as EMG or brain activation (Basalp et al., 2021). In the aspect of human-robot cooperation, an EMG-based admittance control scheme is proposed, which could improve stroke survivors' ankle range of motion and movement stability and have the potential to be applied in clinical rehabilitation training (Zhuang et al., 2021). For implementations of assist-as-need (AAN) by impedance or admittance control (Hussain et al., 2017), it is generally run offline with the gains adaptation to modify the compliance of the robot or sensorless force estimation to model patient's capabilities (Proietti et al., 2017), or realized by adding a fixed model of the patient's limbs dynamics which needs to be manually adjusted for each participant (Wolbrecht et al., 2008). Sensors on the robotic device may provide real-time and objective measures of motor ability, which can help the patients to follow recovery progress in the robotic interventions (Tran et al., 2018), and the sense of the robot assistance could also be accessed from patients.

In the human-robot interaction, the adaptive impedance parameters adjustment enables motion compliance of the assistive robot (Wen et al., 2017). Based on adaptive control, the online impedance estimation can minimize the human-robot interaction force and ensure effective AAN. Different from the lower limb exoskeleton, the end-effector robot for upper limb rehabilitation provides more degree-of-freedoms, but it might result in secondary injury for the extra interactive space beyond the safe range when the paralysis or spasticity leads to the high stiffness of the human joint and inability to react fast (Zhang and Cheah, 2015), and incoordination of workspaces between robot and human might cause potential risks in rehabilitation training, and simply limiting the human-robot interaction force to an appropriate range cannot guarantee the safety of stroke patients in upper limb rehabilitation. To protect the patients in AAN control, the joint space walls are introduced to constrain the movement of the arm joints (Brokaw et al., 2014). With the virtual tunnel technology, the AAN path controller covers the assistance range from a completely passive to active movement without any support (Keller et al., 2013). However, these region control methods rely on position information rather than the motor ability of patients in different rehabilitation stages. With hemiplegia after stroke, most patients suffer from various levels of muscle spasm, increased tension or weakness (Brunnstrm and Englund, 2010). To relieve these symptoms, rehabilitation training can be divided into passive, active and resistive modes according to the category proposed by Brunnstrom (Brunnstrom et al., 2009). In clinical practice, the grades for evaluating the states of a patient's upper limbs are generally calculated by the Modified Ashworth Scale (MAS) (Bohannon and Smith, 1987), which is highly reliable and universal (Tsuji et al., 2002). However, MAS cannot be directly input into the controller (Mehrholz et al., 2005), while the physiologically related parameters such as the muscle contraction force, the joint rotation angle and torque (Yang et al., 2021) are more suitable for control. In particular, methods such as MuscleNET map EMG to kinematic and dynamic biomechanical variables by machine learning for applications in biomechatronic device control (Nasr et al., 2021). There are also exoskeleton AAN control systems using motor ability as input, such as the wearable exoskeleton controlled by surface Electromyography (sEMG) for upper limb power assistance (Liu et al., 2021). Compared with other types of input signals, physiological signals such as sEMG can be detected before and during the actual movement of the patients, which is more convenient to interpret the motion intention and motor ability of the patients as the input of the controller. However, most studies focus on the performance metrics like movement time (Basalp et al., 2021), while the safe human-robot interaction space by sEMG has not been fully investigated. To ensure safe and effective AAN control, the machine learning results of EMG should provide the reference for the interaction space reshaping and the assistance level quantification for adapting the engagement of the patients in real-time.

In this work, a human-robot interaction space reshaping method based on assistance level quantification for rehabilitation training was proposed. The contributions and novelties were listed below.

1. The experimental protocols were designed to quantify the motor ability of patients in stroke rehabilitation. Different from the general AAN robotic rehabilitation training, the designed protocols covered the active, passive and resistive rehabilitation modes, and the training sessions were graded from the perspective of the progress of rehabilitation training, which provided the basis of subdividing the motor ability for safe AAN control by interaction space reshaping.
2. Facing the progress of the patients in stroke rehabilitation, the mapping between the level of motor ability and the training session was established, and the output of the classifier was used for reshaping the interaction space with the spherical virtual boundary. Considering the safe online adjustment, this paper also introduced a Long Short-Term Memory network with Kalman Filter (LSTM-KF) model to predict the perceived torques to provide the gain modification in the AAN control. For more complicated motor tasks related to ADL, it was also helpful to recognize the motor ability of the basic movement in each training session.
3. From the perspective of human-robot interaction, the normalized mean perceived torque (NMPT) and the assistance level were related, which provided further quantification of the motor ability. The variation of the assistance level was analyzed, which characterized the needs of the patients in AAN control by sessions and revealed their engagement in different rehabilitation stages.

This paper consists of four parts, mainly including the following sections: Section 2 is the method used in this research, introducing the experiment protocols for sEMG and kinematic data acquisition, the method of data preprocessing, the establishment of classification and regression models and the AAN control for interaction space reshaping. Section 3 presents the experimental results followed by a discussion about assistance level quantification, and Section 5 concludes the paper.

## 2. Methods

**Figure 1:**
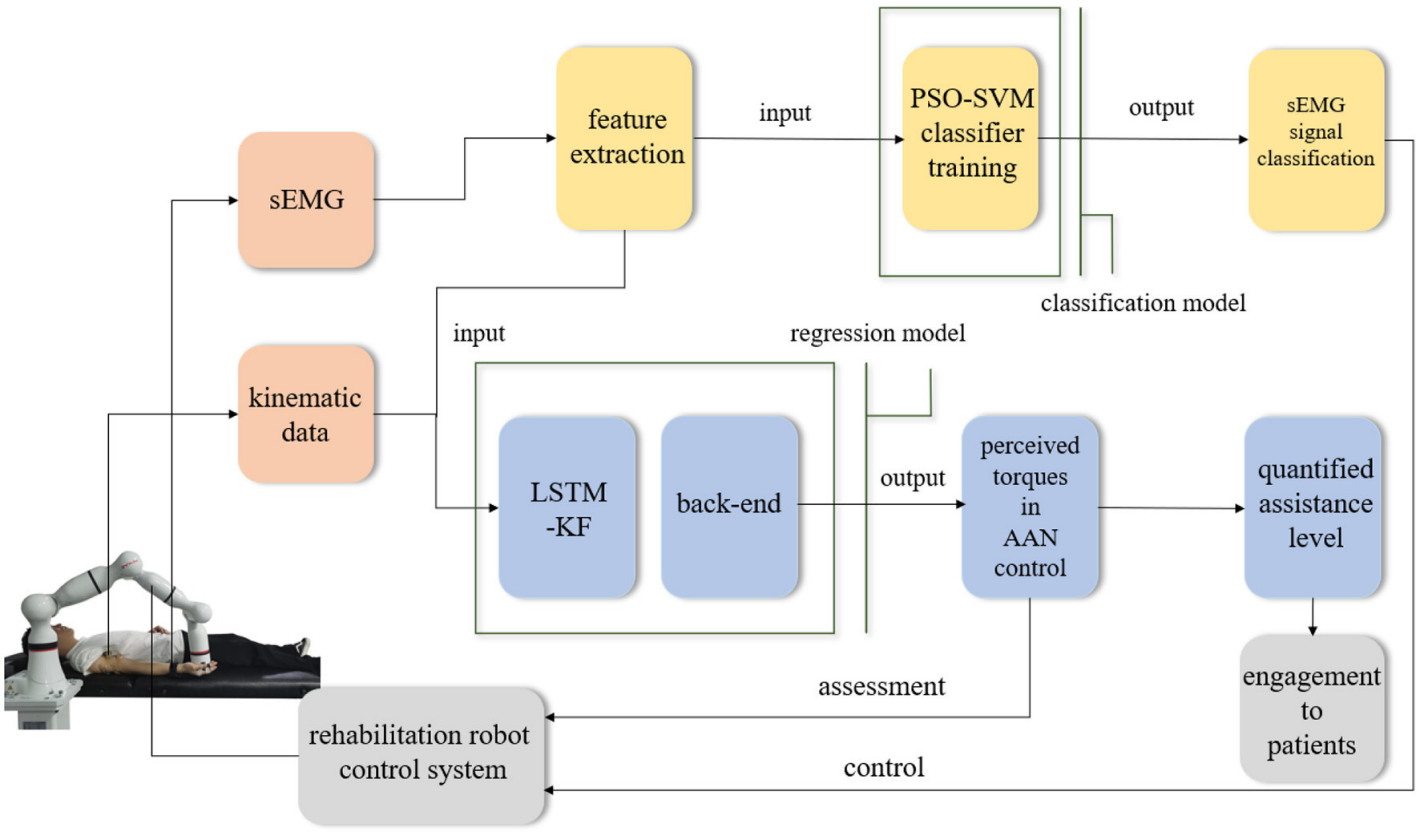
The interaction process between the stroke patient and the rehabilitation robot.

In different rehabilitation stages of stroke, the corresponding rehabilitation training strategies should be designed. To adapt to the different rehabilitation stages of patients and achieve AAN control, the rehabilitation robot needs to sense the muscle state and recognize the rehabilitation stage. It needs a classification algorithm to distinguish different recovery states, and a regression algorithm to obtain the perception of the assistive torque from the robot during continuous training sessions. Therefore, the Support vector machine with Particle Swarm Optimization (PSO-SVM) classifier and LSTM-KF regression models were trained by the sEMG and kinematic data from the participants as the input of AAN control. The complete interaction process between the stroke patient and the rehabilitation robot is shown in Figure 1.

### 2.1. Experiment protocols

It mainly used the following equipment to collect experimental data. An 8-channel sEMG system (BTS FreeEMG300, Italy) was mounted to collect electrical activity of the biceps and triceps which are two important muscles for upper limb movement. At the same time, the participant wore a motion capture sensor on the wrist to measure the angles of rotation of the participant's arm from an initial horizontal position to an arbitrary position on the motion trajectory, and then the first-order and second-order differential data of the angles were calculated. The robot arm (ROKAE Mate ER3 Pro) was used for rehabilitation training experiments of flexion and extension movements of upper limbs.

A total of 10 young males aged 19–25 years were enrolled, all right-handed, healthy, with no known neurological or psychological disorders, and no use of psychotropic drugs or any drugs affecting the central nervous system. In the off-line experiment stage, we enrolled one or two participants to experiment each day. After data collection, we conducted the training of classification models and regression models, and collected a total of 10 participants' offline experimental data. After that, the same 10 participants attended online experiments on another day. The participants were asked to take a good rest the day before the experiments. Each participant was told the protocols and was enrolled in the experiments after receiving verbal consent. The protocol studied was approved by the local ethics committee and implemented following the Declaration of Helsinki.

Referring to the rehabilitation training in clinical therapy, the horizontal rehabilitation bed was used in the collecting data during the experiment, and the representative experimental protocol was designed according to the different stages of actual rehabilitation. Due to the influence of different neural pathways and motor dysfunction, stroke patients have the lower muscle strength and poorer limb coordination. At the beginning of the experiments, we adjusted the initial state of the participants through EMG data collected by the sEMG device to determine their muscle conditions between the states and relaxation and contraction. In the early stage of rehabilitation, the muscle has not enough strength to move, and it improves with the progress of rehabilitation training. According to the rehabilitation process, it divided the rehabilitation training experiment into three stages, which are passive, active and resistive training stages. The passive training required the participant to stay relaxed throughout the session to simulate the completely floppy state and complete flexion and extension arm movement driven by the movement of the robot, and the sEMG amplitude of participants was used to judge whether they remained passive. In general, participants were required to keep their sEMG amplitude below 200 μv during passive training. Due to the individual difference in muscle strength, the value 200 μv could be adjusted slightly for each participant in the experiment. In the active training, the participants needed to simulate that the upper limbs had recovered to a certain extent, could apply force and complete the flexion and extension arm movement with the assistance of a small amount of robot arm. Finally, in resistive training, participants were required to apply their force in the opposite direction of the robot's movement. In other words, when the robot raises upward, participants needed to follow it with downward force. For the downward movement, they needed to apply upward force during the motion, so as to achieve the strengthening purpose by resisting the robot. Next, the above training sessions were categorized according to the patients' motor ability in each stage. There were seven types of experimental protocols: complete passive training (PT), passive training with slight motor ability (PT+), active training with weaker motor ability (AT-), normal active training (AT), active training with stronger motor ability (AT+), resistive training with weaker motor ability (RT-) and resistive training (RT).

The experiment was divided into three steps: First of all, with the assistance of the robot arm, data collection of the above seven experimental protocols was completed off-line to obtain two groups of sEMG data of biceps and triceps, and three groups of kinematic data of joint angle with the first-order and the second-order differences of them. Then, the sEMG data was used as the training set to train the classification algorithm and get the classification model. Meanwhile, the sEMG data and kinematics data were used as input signals to complete the training of the regression model. Next, in the online condition, the participants were asked to perform 7 experimental protocols, and the corresponding rehabilitation training sessions were classified according to the generated model to provide the control and adjustment signals, and by the corresponding regression model, the interaction space was modified and the perceived torque which relates to the assistance level of the robot.

**Figure 2:**
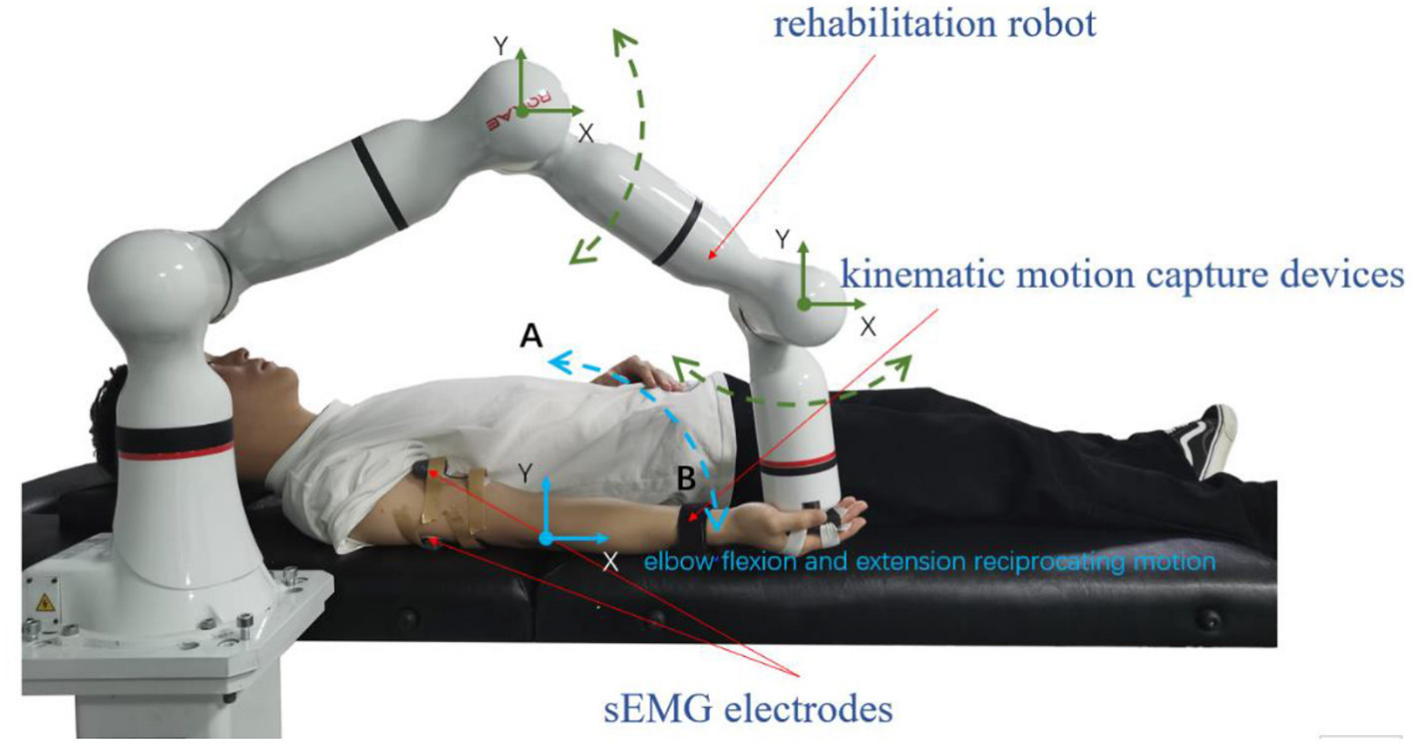
The experiment installation of the rehabilitation training.

During data collection, the skin of the participants was cleaned first, and sEMG electrodes and motion capture sensor were placed on the arms of the participants to identify muscle activation and joint rotations. An elastic binding band was mounted on the end effector of the robot so that the palm of the participant could be connected on it compliantly (Zhang et al., 2020). The experiment installation position and the trajectory of flexion and extension arm movement are shown in Figure 2. The robot in the experiment had built-in joint angle position sensors on each joint. Then, the 7 × 1 joint Angle vector was converted into the coordinates of the end-effector in Cartesian space by the underlying packaged real-time Jacobian matrix calculation program, and the coordinate value was stored in real-time. When the palm of the participant was connected to the end-effector with the binding band, the actual training trajectory of the patients was obtained. In the offline training data collection experiments, the participants lay flat on the bed with their arms and thighs parallel to the ground. With the assistance of the rehabilitation robot, they carried out the rehabilitation training of flexion and extension movements of upper limbs according to the designed seven experimental protocols. As shown in Figure 2, in each experimental protocol, 20 s of flexion movements were performed first, and then 10 s of rest after reaching the highest point A, then 20 s of stretching movement and 10 s of rest after reaching the lowest point B. The above actions were regarded as one trial, which took 60 s in total. Each session included two trials, which took 120 s in total, and the rest between the two sessions lasted 3 min. Each participant needed 32 min to complete the seven experiment protocols. The safety of the participants must be ensured during the experiment. If the patients appear palpitation, dizziness and other discomforts, they should immediately stop the experiment. The process of the online experiment followed that of the offline experiment. The rehabilitation training sessions were classified when the participants applied force according to different experiment protocols, and the corresponding control signals were transmitted to the robotic arm so that it could reshape the interaction space to different recovery states for safety. At the same time, with the output of the regression model, the gain coefficient was calculated by the perceived torques of the participants in the human-robot interaction for AAN control, by which the assistance level is quantified according to the engagement of patients.

### 2.2. Online experiment system construction

**Figure 3:**
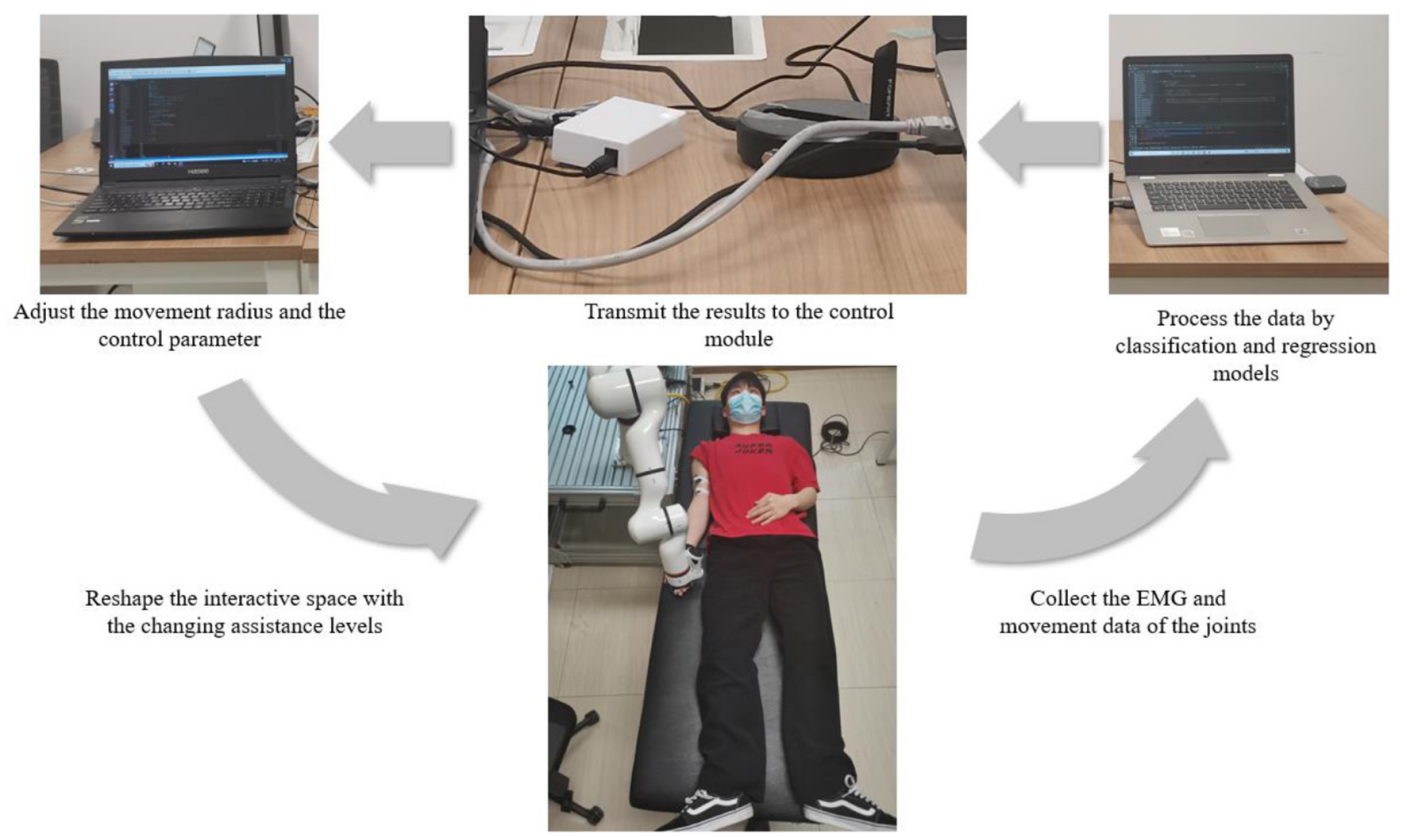
The working process of the online experiment.

To verify whether the assistance method of rehabilitation training proposed in this paper can realize the recognition of different recovery states in the actual rehabilitation process and whether the AAN control could be implemented with the changing assistance levels in continuous training sessions, it carried out an online experiment design on the basis of offline experiment, with the online system including patient, data processing and control modules, as shown in Figure 3.

In the online experiment, the participants also completed the rehabilitation training of upper limb flexion and extension with the assistance of the robotic arm following seven experimental protocols. The difference is that the data collected in the online experiment will be processed in real time. After processing by classification and regression models, the recognition of the current rehabilitation state of the participants and the interaction torque in AAN control could be obtained respectively. After that, the results were transmitted to the control module through the switch, to achieve the following two purposes. (i) the movement radius of the robotic arm could be adjusted according to the current training sessions of the participants so that targeted rehabilitation training could be conducted according to the recovery states. Patients with unstable strength, as determined by sEMG, have not fully recovered from peripheral nerve injury in the upper limb and have not fully activated peripheral muscle control (Li et al., 2021), and the robotic arm can adjust the interaction space according to the classification results to play a protective role; (ii) At the same time, according to the perceived torques obtained by the regression model, the gains of the robot arm control can be modified, which implements the AAN control and has the relationship to the quantification of the assistance level in rehabilitation training.

### 2.3. EMG data preprocessing and machine learning

To achieve real-time tracking of the motor ability of patients and adjust the control strategies of rehabilitation robots according to rehabilitation stages, this paper uses the data of different rehabilitation training experiments of each participant to conduct the training of classification and regression models. Support vector machine (SVM) has the advantages of simplicity, robustness, high precision and strong generalization ability. In this paper, SVM was utilized for the session classification model training. At the same time, it is found that (Greff et al., 2017), compared with the regression model trained by traditional parameter identification methods, the accuracy of joint torque estimation is significantly improved in the regression model with the application of Long Short-Term Memory network (LSTM), and the error value of the model is also reduced. Therefore, an LSTM-based training regression model was introduced to predict the perceived torques in AAN control from the patient, which provided the input of the feedback gain modification of the rehabilitation robot and the quantification of the assistance level by perceived torques to reveal the engagement of patients to the AAN control.

The sEMG data was filtered to remove noise and keep the useful information unchanged. The valid frequencies of the sEMG original signal mainly locate at 0–500Hz, and the frequency range of noise generated by power-line interference, electrode movement and motion artifacts are all below 20Hz. A fifth-order Butterworth bandpass filter (20–500Hz) was used to process the raw sEMG signal. Meanwhile, to extract the active EMG signal and eliminate the useless information in the rest states, the EMG data from the biceps and triceps channels were intercepted. In addition, the threshold value worked for eliminating inactive data in all channels of each participant. The threshold was set as 70%, and the data with the top 70% amplitude will be retained and the remaining data will be removed.

#### 2.3.1. PSO-SVM classifier

SVM is a binary classifier that finds an optimal hyperplane in the feature space, with the distance in the feature space which maximizes the distances between the features of two groups of sample data, to generate the classification model. Although SVM has strong generalization and classification ability, it is sensitive to noise, and the EMG signal is also weak and easy to be influenced by noise interference, which leads to the difficulty of data processing. In addition, the more sample data of SVM model training, the more constraints are required, which will lead to the reduction of the training speed and the deterioration of the stability of the model. In this paper, the performance of SVM was improved by optimizing its parameters. The main optimized parameters are penalty parameter *C* and kernel parameter σ. Particle Swarm Optimization (PSO) is a swarm intelligence algorithm designed by simulating the predation behavior of birds. Its purpose is to make all particles find the optimal solution in the multidimensional search space. Because of its advantages such as simple principle, easy implementation and fast convergence speed, it has been used in many studies to optimize SVM parameters in recent years. Therefore, this paper also utilized PSO to optimize SVM parameters here. In this section, the fundamentals of the SVM algorithm and explains the PSO algorithm were introduced, then the two algorithms for the application in the sEMG classification were combined.

SVM is mainly divided into linearly separable and non-separable cases. In the former case, for sample data (*x*_*i*_, *y*_*i*_), *i* = 1, 2......, *n, x* ∈ *R*^*d*^, *y* ∈ |− 1, + 1|, it can find the optimal hyperplane *w*^*T*^*X* + *b* = 0, and the sample data to that hyperplane with the distance of 2/‖*w*‖, therefore classification problem is converted into the constrained optimization problem:


(1) $$\begin{array}{l} \min\frac{1}{2}{\left\|w\right\|}^{2} \end{array}$$

(2) $$\begin{array}{l} s.t.\;y_{i}\left(wx_{i}+b\right)\geq 1 \end{array}$$

In the non-separable case, sample classification cannot be done with a straight line, so it is necessary to allow the existence of a few sample data points that do not meet the requirement of *y*_*i*_ (*wx*_*i*_ + *b*) ≥ 1, and the loose variable ξ_*i*_ ≥ 0 (i = 1, 2..., n) and penalty parameter C is introduced, then the classification problem can be modified to:


(3) $$\begin{array}{l} \min\frac{1}{2}\|w|{|}^{2}+C\overset{n}{\underset{i=1}{\sum }}\xi_{i} \end{array}$$

(4) $$\begin{array}{l} s.t.\;y_{i}\left(wx_{i}+b\right)-1+\xi_{i}\geq 0 \end{array}$$

By adjusting the values of ξ_*i*_ and *C*, invalid data points can be removed to avoid overlearning. The formula for solving the hyperplane is as follows:


(5) $$\begin{array}{l} f\left(x\right)=sgn\left[\sum_{i=1}^{n}\alpha_{i}*y_{i}k\left(x_{i},x\right)+b*\right] \end{array}$$

Where the kernel function is a radial basis expression:


(6) $$\begin{array}{l} k\left(x_{i},x\right)=\exp\left(-\frac{\|x_{i}-x|{|}^{2}}{2\sigma^{2}}\right) \end{array}$$

Next, the PSO algorithm is used to optimize the SVM parameters. The principle of the PSO algorithm is to constantly update its own speed and position by searching the individual and the overall optimal values. After several iterations, the optimal combination of target parameters can be obtained. The specific expression is as follows:


(7) $$\begin{array}{ll} v_{t+1}=\omega v_{t} & +c_{1}r_{1}\left(pbest_{t}-x_{t}\right)+c_{2}r_{2}\left(gbest_{t}-x_{t}\right) \end{array}$$

(8) $$\begin{array}{l} x_{t+1}=x_{t}+v_{t+1} \end{array}$$

Where, *v*_*t*_ represents the speed of iteration at time *t*, *v*_*t*+1_ represents the speed of iteration at time *t* + 1, *x*_*t*_ represents the position of iteration at time *t*, *x*_*t*+1_ represents the position of iteration at time *t* + 1, ω is the inertia weight, *c*_1_ and *c*_2_ are learning factors, *r*_1_ and *r*_2_ are 0–1 random numbers. *pbest*_*t*_ is the position of the individual extreme point when the particle iterates at time t, and *gbest*_*t*_ is the position of the whole extreme point when the particle iterates at time *t*.

PSO serves for the parameter optimization of SVM. The steps of the algorithm mainly include: the samples were divided into training and test sets, and the normalization was carried out. The particle swarm parameters were initialized and the particle fitness was calculated. According to particle fitness, the position of the individual extreme point and that of the whole extreme point were found. With the updated particle velocity and position, the classifier was generated by the training set to determine whether the accuracy was optimal. If so, the penalty parameter *C* and the kernel parameter σ were set as output; if not, the particle fitness was recalculated and the iteration continued.

The PSO-SVM method was used to classify sEMG data from the three rehabilitation stages. First, the active part of the data was intercepted, and stratified sampling was carried out to obtain a data set enough to reflect the overall trend of the original data. After that, feature extraction was carried out on the obtained data set using the time-domain method. The main time-domain features extracted were the mean absolute value (MAV, reflecting the overall muscle activation state), wavelength length (WL, reflecting the complexity of waveform), zero crossing number (ZC, reflecting the complexity of sample), root mean square (RMS), autoregressive coefficient (AR, reflecting the data transformation state). After feature extraction, each sEMG data fragment could be replaced by the extracted feature vector. After all the data of each major stage was preprocessed, the corresponding label representing the session to which the data belongs could be used for model training based on PSO-SVM.

#### 2.3.2. Regression model based on LSTM-KF

LSTM is a special type of Recurrent Neural Network (RNN), which is used to process time series information with longer intervals. Compared with RNN, LSTM adds input thresholds, forgetting thresholds and output thresholds, and the weight will always change in the process of self-cycling. Considering that LSTM is easily affected by the length of data series (Simo et al., 2019), and the Kalman filter is capable of optimizing the internal state of the system according to the observed value of the system, it can be combined with LSTM to improve the prediction accuracy. In this paper, the collected kinematic data is processed by the Kalman filter, and its prediction process is embedded in the information memory process of LSTM to build the LSTM-KF model and obtain the best observed value.

LSTM controls and protects the cell state by setting different threshold combinations, and screens and removes the information in the time series. The input of LSTM includes the cell state (*c*_*t*_), the hidden layer input (*h*_*t*−1_) and the current input (*x*_*t*_). It works by receiving the current input *x*_*t*_ and the output *h*_*t*−1_ transmitted from the previous state. Through the splicing of different activation functions, different threshold assignments are completed to obtain the output value after information screening. The mathematical expressions of the forgetting threshold, input threshold, cell status update and output threshold are as follows:

Threshold of forgetting:


(9) $$\begin{array}{l} f_{t}=\sigma \left(W_{f}\left[h_{t-1},x_{t}\right]\right) \end{array}$$

Input threshold:


(10) $$\begin{array}{l} i_{t}=\sigma \left(W_{i}\left[h_{t-1},x_{t}\right]\right) \end{array}$$

(11) $$\begin{array}{l} \tilde{C_{t}}=\tanh\left(W_{C}\left[h_{t-1},x_{t}\right]\right) \end{array}$$

Cell status updating:


(12) $$\begin{array}{l} C_{t}=f_{t}\;*\;C_{t-1}+i_{t}\;*\;\tilde{C_{t}} \end{array}$$

Output threshold:


(13) $$\begin{array}{l} o_{t}=\sigma \left(W_{o}\left[h_{t-1},x_{t}\right]\right) \end{array}$$

The resulting output:


(14) $$\begin{array}{l} h_{t}=o_{t}*\tanh\left(C_{t}\right) \end{array}$$

In the above equations, σ represents the sigmoid function; *W* is the weight matrix of different thresholds; *o*_*t*_,*i*_*t*_,*f*_*t*_ are the values between 0 and 1 obtained by sigmoid function conversion, indicating the gate state. $\tilde{C_{t}}$ as tanh function conversion of input data has the value between −1 and 1.

As mentioned above, the performance of the trained LSTM model is easily affected by the length of the time series. To reduce this impact, the Kalman filter (KF) and LSTM are combined in this paper. KF can predict the uncertain dynamic system with good anti-interference. Assume that the state of the system at time *t* is *X*_*t*_, the covariance matrix of the prediction error at time *t* is *R*_*t*_, and the covariance matrix of the system state and prediction error of the previous state is *X*_*t*−1_ and *R*_*t*−1_ respectively. For KF, according to mean square error (MSE), the best estimate of time t is ${\hat{X}}_{t}$. Considering external control variables and environmental factors, the corrected result is as follows:


(15) $$\begin{array}{l} {\hat{X}}_{t}=F_{t}{\hat{X}}_{t-1}+B_{t}\vec{u_{t}} \end{array}$$

(16) $$\begin{array}{l} R_{t}=F_{t}R_{t-1}F_{t}^{T}+Q_{t} \end{array}$$

Where *F*_*t*_ represents the prediction process of KF, *B*_*t*_ is the control matrix, $\vec{u_{t}}$ is the control vector, and *Q*_*t*_ is the covariance matrix of environmental noise. According to the observation data of *t* time, it can be on behalf of the prediction part of the gaussian distribution $\left(\mu_{0},\Sigma_{0}\right)=\;\left(H_{t}{\hat{X}}_{t},H_{t}R_{t}H_{t}^{T}\right)$ and measure the part of the gaussian distribution (μ_1_, Σ_1_) = (Ŷ_*t*_, *P*_*t*_), where *H*_*t*_ is the observation matrix, Ŷ_*t*_ is the observed value, and *P*_*t*_ is the observed covariance matrix. With the two multiplied distributions, it can get through the KF by the updated optimal estimate distribution $\left(\mu^{{\;}^{\prime }},\Sigma^{{\;}^{\prime }}\right)=\;\left(H_{t}{{\hat{X}}_{t}}^{{\;}^{\prime }},H_{t}{R_{t}}^{{\;}^{\prime }}H_{t}^{T}\right)$ :


(17) $$\begin{array}{l} \left(\mu^{{\;}^{\prime }},\Sigma^{{\;}^{\prime }}\right)=\left(\mu_{0}+K\left(\mu_{1}-\mu_{0}\right),\Sigma_{0}-K\Sigma_{0}\right) \end{array}$$

After plugging in the data, it gets:


(18) $$\begin{array}{l} {{\hat{X}}_{t}}^{\prime }={\hat{X}}_{t}+{H_{t}}^{-1}K\left({Ŷ}_{t}-H_{t}{\hat{X}}_{t}\right)={\hat{X}}_{t}+K^{\prime }\left({Ŷ}_{t}-H_{t}{\hat{X}}_{t}\right) \end{array}$$

(19) $$\begin{array}{l} {R_{t}}^{\prime }=R_{t}-{H_{t}}^{-1}KH_{t}R_{t}=R_{t}\left(I-{H_{t}}^{-1}KH_{t}\right)=R_{t}\left(I-K^{\prime }H_{t}\right) \end{array}$$

Where the optimal estimate (${{\hat{X}}_{t}}^{\prime }$) and the prediction error covariance matrix (${R_{t}}^{\prime }$) can get updated after KF. K is the Kalman gain matrix, *K*′ is the updated Kalman gain:


(20) $$\begin{array}{l} K=\Sigma_{0}{\left(\Sigma_{0}+\Sigma_{1}\right)}^{-1}=H_{t}R_{t}H_{t}^{T}{\left(H_{t}R_{t}H_{t}^{T}+P_{t}\right)}^{-1} \end{array}$$

(21) $$\begin{array}{l} K^{{\;}^{\prime }}={H_{t}}^{-1}K=R_{t}H_{t}^{T}{\left(H_{t}R_{t}H_{t}^{T}+P_{t}\right)}^{-1} \end{array}$$

The memory and forgetting function of LSTM and the anti-interference prediction function of KF are combined to construct the LSTM-KF model. The mean value of the system state at the last moment (${\hat{X}}_{t-1}$) is input into LSTM to obtain the current mean value of the system state (${\hat{X}}_{t}$). After processing by the KF algorithm, the optimal estimated mean value (${{\hat{X}}_{t}}^{{\;}^{\prime }}$) is obtained.

**Figure 4:**
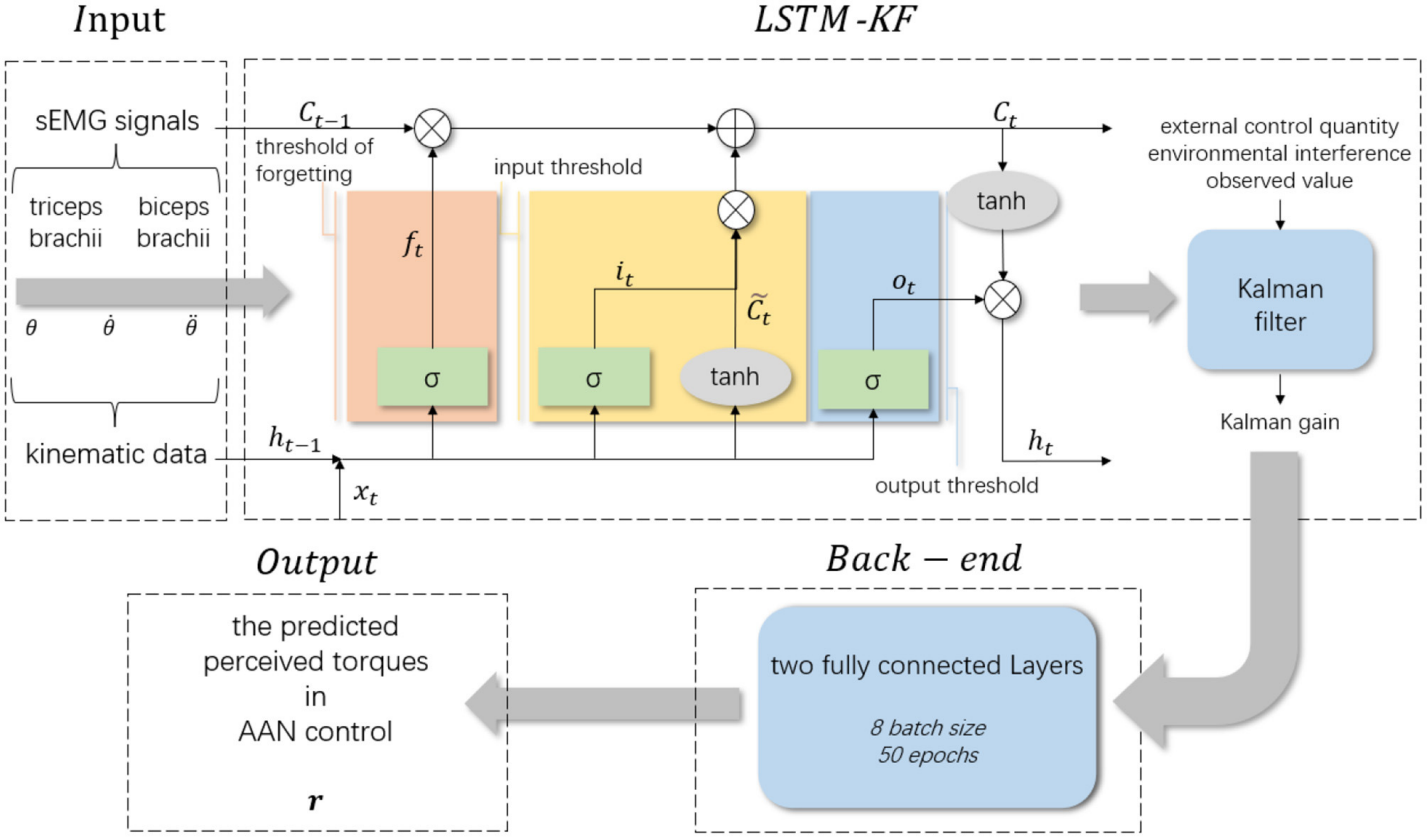
The framework of the regression model.

In this section, the LSTM-KF-based neural network model was used for the regression of the data from the experiment to predict the perceived torques for AAN control and be used to quantify the assistance level of the rehabilitation robot. The framework of the regression model is shown in Figure 4, which is divided into four parts: input layer, LSTM-KF model, back-end fully connected layer and output layer. The input layer received sEMG signals and kinematic data collected from the participants in various rehabilitation states. The sEMG signal contained two channels corresponding to the biceps brachii and triceps brachii. The kinematic data contained three channels corresponding to joint Angle (θ), the first-order difference of joint Angle ($\dot{\theta }$) and the second-order difference of joint Angle ($\ddot{\theta }$) respectively. The dimension of the received data was 5 and the dimension of the hidden layer was 64. Since the sampling frequencies of sEMG signal acquisition equipment and kinematic signal acquisition equipment were different, the sampling rate of the sEMG signal was 1000Hz, and the sampling rate of kinematic data was 60Hz, which needed to be down-sampled before the neural network model was input, and then sEMG signal and kinematic data were fused. Thus, a data matrix with a size of about 13,000 × 5 was obtained, which was a total of 13,000 data points with 5 eigenvalues for each data point. Next, it sliced the data matrix and taked 200 data points as a group, with the characteristic values of the first 199 data points as a single input and the moment value of the 200th point as a label, so the length of the sliding window was 200, and the step value was 10. At the back end, there were two fully connected layers, and the batch size was set to 8 and the epochs to 50. There was an additional dropout layer between the two to avoid overfitting, and the final output was the predicted 1-dimensional data of the perceived torque value of the participants.

### 2.4. Assist-as-needed control

Rehabilitation training is a typical human-computer interaction scenario. Stroke patients often experience unstable muscle strength, which makes them highly susceptible to a sudden drop of the limbs caused by muscle fatigue or spasm during rehabilitation training, resulting in secondary injury. Therefore, it is very important to ensure the safety of users while improving the rehabilitation effect by using AAN control strategy. To meet the safety requirements, the level of assistance should match the patient's motor ability (Zhang et al., 2020). The proposed ANN control strategy was achieved through the coordination of different sub-functional modules for safety aims. Firstly, the patient's EMG signal was analyzed using SVM-PSO and LSTM-KF to recognize their motor ability, which would then serve as the basis for adjusting the control parameters of the region controller by artificial potential fields. With the controller, the interaction space reshaping was implemented by matching the patient's motor ability safely when fatigue or spasm occured during the training stages and sessions.

#### 2.4.1. Robot kinematics and dynamics

In rehabilitation training, which emphasizes human-robot interaction, the desired path for the end-effector of the robot is usually specified in task space. Thus, let *X* ∈ *represent*ents the position and orientation vector of the end-effector in the Cartesian space.


(22) $$\begin{array}{l} X=f\left(q\right) \end{array}$$

where *X* ∈ *R*^*n*^ represents the position vector of the robot's end-effector in the Cartesian space, *q* ∈ *R*^*n*^ is a *n* × 1 joint angle vector indicating the pose state of the robot in joint space and *f* (.) ∈ *R*^*n*^ → *R*^*n*^ represents the mapping from joint space to task space. Then take the derivation of Equation (22)


(23) $$\begin{array}{l} \overset{.}{X}=J\;\left(q\right)\overset{.}{q} \end{array}$$

Where $\overset{.}{X}$ is the velocity vector of the end-effector in the Cartesian space and $\overset{.}{q\;}$ means the joint velocity vector in joint space, *J* (*q*) ∈ *R*^*n*×*n*^ is the Jacobian matrix of mapping the velocity from joint space to task space.

In the scenario of interactive rehabilitation training, torque control is more suitable. Thus, the rehabilitation robot's dynamics are described as follows.


(24) $$\begin{array}{l} M\left(q\right)\overset{..}{q}+C\left(q,\overset{.}{q}\right)\overset{.}{q}+G\left(q\right)=\tau \end{array}$$

*M* (*q*) ∈ *R*^*n*×*n*^ is the inertia matrix that is symmetric and positive definite, $C\;\left(q,\overset{.}{q}\right)\overset{.}{q}\in R^{n}$ represents the centrifugal force and the Coriolis force vector, *G* (*q*) ∈ *R*^*n*^ denotes a gravitational force vector, and τ ∈ *R*^*n*^is the joint control torque input. Inertia matrix *M* (*q*), centrifugal force and the Coriolis force vector $C\;\left(q,\overset{.}{q}\right)$ have the following relationship.


(25) $$\begin{array}{l} {\overset{.}{q}}^{T}\left(\overset{.}{M}-2C\right)\overset{.}{q}=0 \end{array}$$

#### 2.4.2. Region controller design for space shaping

**Figure 5:**
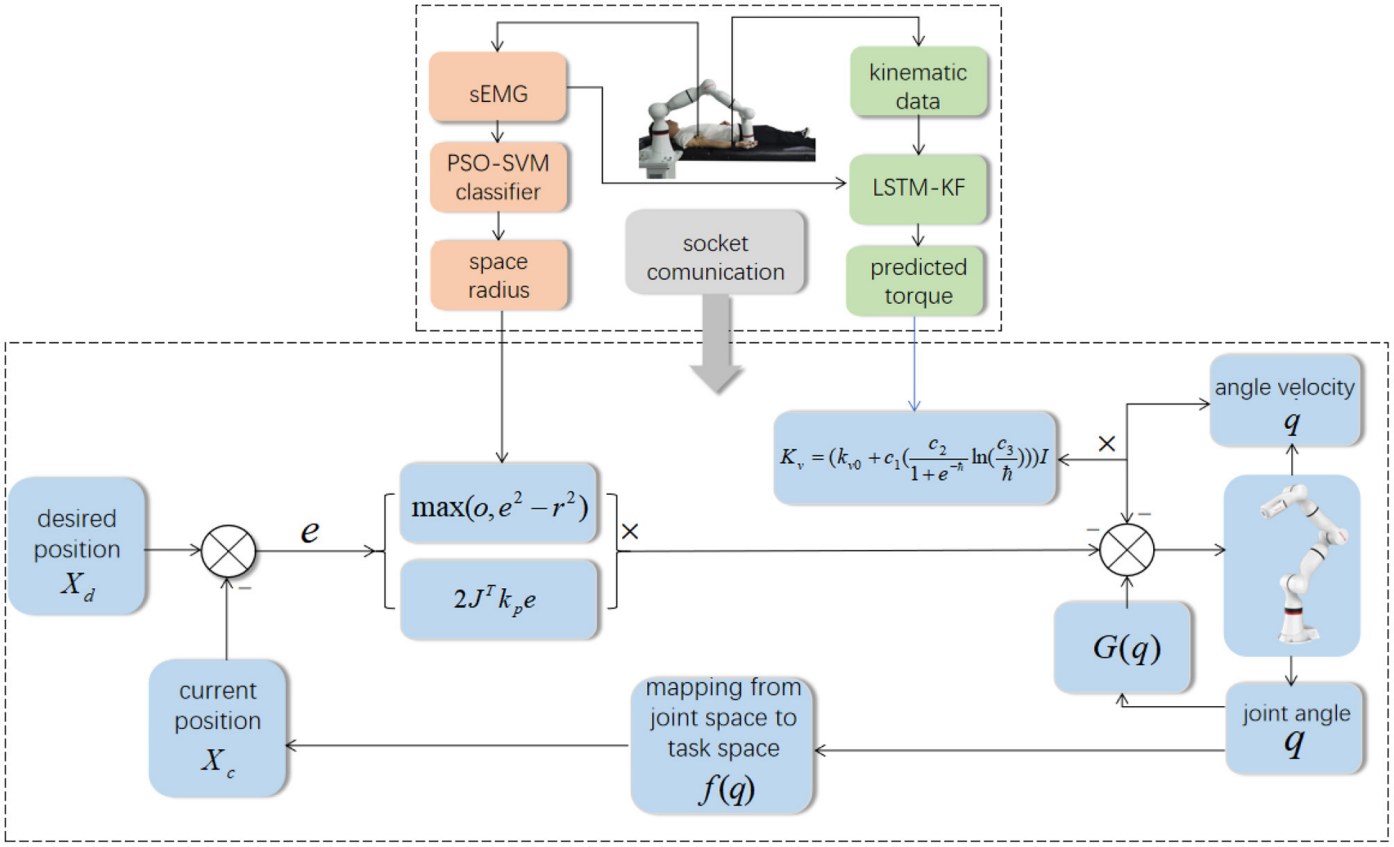
The framework of AAN control.

Different from the conventional trajectory tracking control strategy for the robot, it is expected that patients can have more engagement in clinical rehabilitation training and can adjust the control parameters online according to the patient's rehabilitation sessions in real time to meet the rehabilitation needs of patients. Therefore, an AAN control strategy based on the patient's EMG signals and kinematic data for online adjustment is proposed as Figure 5, which ensures safe and effective training by human-robot interaction space reshaping and control parameter adjustment online.

For the fixed-point position control, the control goal is to make the task-space position error *e* = *X*_*c*_ − *X*_*d*_ converge to zero eventually, where *X*_*c*_ and *X*_*d*_ denotes the current position and desired position respectively. To meet this goal, an error-based potential function *F*(*e*) is specified as


(26) $$\begin{array}{l} F\left(e\right)=\frac{1}{2}ek_{f}e^{T} \end{array}$$

note that *k*_*f*_ is a constant gain. Get the gradient of *F*(*e*) as following.


(27) $$\begin{array}{l} \frac{\partial F\;\left(e\right)}{\partial e}=k_{f}e \end{array}$$

note that *e* approaches zero as the gradient of potential function close to zero. Therefore, to accomplish the control goal, a control term containing a negative gradient of potential function can be considered in the control law to continuously drive the robot arm close to the desired position until the error *e* is zero.

Different from fixed-point position tracking, considering that the clinical application of rehabilitation training does not require high accuracy in tracking performance, and to ensure the safety of patients and improve the positivity of rehabilitation training of patients, it considers a region tracking controller, which means as long as the end-effector of the rehabilitation robot reaches the specified region, and the control scheme is executed and the control objective is fulfilled. In this work, it considers a spherical space defined as following.


(28) $$\begin{array}{l} \Omega =\left\{X|g\;\left(X\right)=\;{\left(x-x_{0}\;\right)}^{2}+\;{\left(y-y_{0}\right)}^{2}+\;{\left(z-z_{0}\right)}^{2}-r^{2}\leq \;0\right\} \end{array}$$

where ${\left[x_{0},y_{0},z_{0}\right]}^{T}\in R^{3}$ is the center of the spherical region, *X* = [*x, y, z*]^*T*^ ∈ *R*^3^ is the current position of the end-effector of the robot, *r* is the radius of the spherical area. *g*(*X*):*R*^3^ → *R* is the target function.

The size of the defined region can be adjusted based on the classification results of EMG signals and kinematic data collected from the participants. Considering the actual requirements of rehabilitation training, the rehabilitation training experiment is divided into three different stages as mentioned above: (i) Passive training mode; (ii) Active training mode; (iii) Resistive training mode. The three stages can be further subdivided into seven different protocols according to actual safety requirements: complete passive training (PT), passive training with slight motor ability (PT+), normal active training (AT), active training with weaker motor ability (AT-), active training with stronger motor ability (AT+), resistive training (RT) and resistive training with weaker movement ability (RT-). Therefore, the radius of the free-movement space for different protocols is as follows.


(29) $$\begin{array}{l} r=\{\begin{array}{c} r_{a},PT\\ r_{b},PT\text{+}\\ r_{c},AT-\\ r_{d},AT\\ r_{e},AT+\\ r_{f},RT-\\ r_{g},RT \end{array} \end{array}$$

Where *r*_*a*_, *r*_*b*_, *r*_*c*_, *r*_*d*_, *r*_*e*_, *r*_*f*_, *r*_*g*_ are seven discrete values, considering the different motor abilities with safety requirements in training sessions of different protocols, the radius value was set as *r*_*e*_ > *r*_*d*_ > *r*_*c*_ > *r*_*g*_ > *r*_*f*_ > *r*_*b*_ > *r*_*a*_.

To make the control objectives clearer, the rewritten formula (28) based on error *e* is as follows.


(30) $$\begin{array}{l} g\;\left(e\right)=\|e|{|}^{2}-r^{2} \end{array}$$

(31) $$\begin{array}{l} e=X_{c}-X_{d} \end{array}$$

where${\;X}_{c}\in R^{n}$ represents the current position vector of the robot's end-effector, and $X_{d}\in R^{n}$ denotes the desired position vector of the robot's end-effector in the Cartesian space. *g* (*e*):*R*^*n*^ → *R* is the objective function of control. The target function is chosen to be continuous and differentiable concerning *e* so that the boundedness of *g* (*e*) assures the boundedness of its first and second partial derivatives. Thus, the potential function in the task space is specified as Equation (32).


(32) $$\begin{array}{l} P\left(e\right)=\frac{k_{p}}{2}\max\;{\left(0,g\left(e\right)\right)}^{2}=\frac{k_{p}}{2}\max\;{\left(0,||e|{|}^{2}-r^{2}\right)}^{2} \end{array}$$

Which means,


(33) $$\begin{array}{l} P\left(e\right)=\{\begin{array}{cc} 0, & g\left(e\right)\leq 0\\ \frac{k_{p}}{2}g{\left(e\right)}^{2}, & g\left(e\right)>0 \end{array} \end{array}$$

Where *k*_*p*_ is a positive coefficient.

Thus, the gradient of the potential function *P*(*e*) is as follows.


(34) $$\begin{array}{l} {\left(\frac{\partial P\left(e\right)}{\partial e}\right)}^{T}=\{\begin{array}{cc} 0, & g\left(e\right)\leq 0\\ k_{p}g\left(e\right){\left(\frac{\partial g\left(e\right)}{\partial e}\right)}^{T}, & g\left(e\right)>0 \end{array} \end{array}$$

The rewritten formula (34) is as follows.


(35) $$\begin{array}{l} {\left(\frac{\partial P\left(e\right)}{\partial e}\right)}^{T}=2k_{p}\max\;\left(0,g\left(e\right)\right)e \end{array}$$

As known from (34), (∂*P* (*e*)/∂*e*)^*T*^ is continuous because *g* (*e*) is close to zero as *X*_*c*_ approaches the boundary of the space defined by formulation (28) and it remains zero when *X*_*c*_ is inside the given space. Based (35), it defines an interactive space tracking controller as Equation (36).


(36) $$\begin{array}{l} \tau =-K_{v}\overset{.}{q}-J^{T}\;\left(q\right)\;{\left(\frac{\partial P\left(e\right)}{\partial e}\right)}^{T}+G\left(q\right) \end{array}$$

The second term in the control scheme (36) contains the negative gradient term of the potential function, which will continuously drive the manipulator to move into the desired region until the gradient value is zero. *G*(*q*) is a *n* × 1 gravity compensation term. And $K_{v}\in R^{n\times n}$ is a positive definite velocity feedback gain matrix.


(37) $$\begin{array}{l} K_{v}=k_{v}I \end{array}$$

*k*_*v*_ is a positive scalar gain changing with the training protocols of the patient and *I* ∈ *R*^*n*×*n*^ represents a unit matrix. The gain *k*_*v*_ should be adjustable to meet the actual engagement needs of patients relating to the perception of assistance levels. Therefore, it establishes the mapping between the gain and the perceived torque that is based on the LSTM-KF regression from sEMG and kinematic data.


(38) $$\begin{array}{l} k_{v}=k_{v0}+c_{1}\;\left(\frac{c_{2}}{1+e^{-\hbar }}\ln\;\left(\frac{c_{3}}{\hbar }\right)\right) \end{array}$$

where *k*_*v*0_ is a positive constant which ensures *k*_*v*_is positive. ℏ means the perceived torque estimated by LSTM-KF with sEMG and kinematic data. *c*_1_, *c*_2_, *c*_3_ are three positive constants.

Thus, it yields the equation of the gain *K*_*v*_.


(39) $$\begin{array}{l} K_{v}=\;\left(k_{v0}+c_{1}\;\left(\frac{c_{2}}{1+e^{-\hbar }}\ln\;\left(\frac{c_{3}}{\hbar }\right)\right)\right)I \end{array}$$

Substituting the (39) into the control law (36).


(40) $$\begin{array}{l} \tau =-\;\left(k_{v0}+c_{1}\;\left(\frac{c_{2}}{1+e^{-\hbar }}\ln\;\left(\frac{c_{3}}{\hbar }\right)\right)\right)I\overset{.}{q}-2J^{T}\;\left(q\right)k_{p}\\ \max\;\left(0,g\;\left(e\right)\right)e+G\;\left(q\right) \end{array}$$

*J*^*T*^ (*q*) is the transpose of the Jacobian matrix.

Then the framework of AAN control is shown below from formulation (40).

## 3. Experiment results

### 3.1. Results of machine learning

The EMG and kinematic data collected were processed by trained classification and regression models. The output of the classification model was applied to the AAN control system, and the rehabilitation robot was controlled according to the motor ability of the patients. The output of the regression model was expressed as perceived torques in AAN control, which would be a basis for quantifying the assistance level of the rehabilitation robot to the patients from the perspective of human-robot interaction. The following are the processing results of classification and regression models.

#### 3.1.1. Results of the classification model

For 10 participants and seven experimental protocols, it used the PSO-SVM algorithm mentioned in Section 2.2.1 to conduct offline experiments and model training. To verify the performance of the classification model, 90% of the data sets were used as the training set and the remaining 10% as the test set. Firstly, all the original data of each participant were preprocessed and segmented. To accurately characterize the rehabilitation status of the participants, the method of threshold detection and the sliding window was adopted to process the data, and the sEMG signals of the patients were mapped to the high-dimensional vector with as less as features loss. The classification accuracy of 10 participants in rehabilitation sessions of passive training, active training and resistive training under offline conditions is shown in Table 1.

**Table 1:** The classification accuracy of 10 participants in three training stages in offline condition.

	**Passive**		**Active**		**Resistive**	
	**Train**	**Test**	**Train**	**Test**	**Train**	**Test**
S1	100.00%	99.65%	100.00%	99.87%	98.95%	99.25%
S2	100.00%	100.00%	99.25%	98.80%	99.70%	99.10%
S3	100.00%	100.00%	99.78%	98.44%	100.00%	100.00%
S4	98.43%	98.59%	99.85%	99.78%	97.62%	96.55%
S5	100.00%	99.54%	100.00%	100.00%	97.17%	96.73%
S6	100.00%	99.76%	100.00%	99.61%	93.14%	94.75%
S7	100.00%	100.00%	99.78%	100.00%	90.95%	90.48%
S8	100.00%	100.00%	100.00%	99.88%	100.00%	99.85%
S9	97.81%	98.54%	98.66%	97.67%	99.85%	99.77%
S10	100.00%	100.00%	95.37%	93.98%	99.52%	99.52%

**Figure 6:**
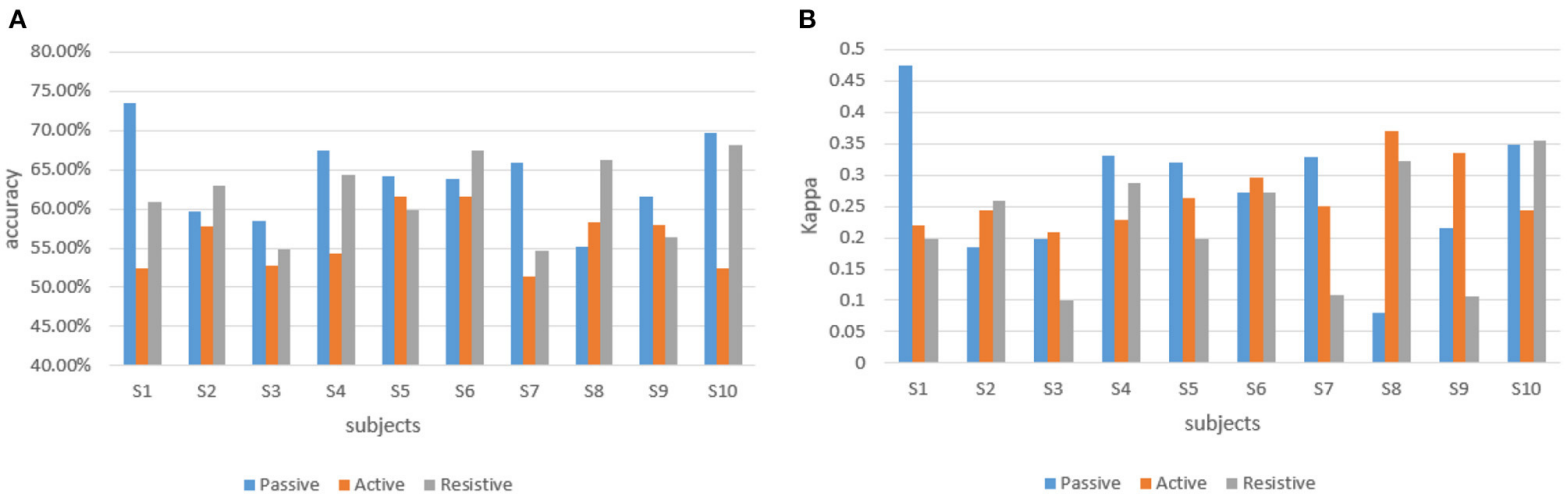
The classification accuracy and Kappa coefficient of 10 participants in three training stages under the online experiment. **(A)** The classification accuracy of 10 participants. **(B)** Kappa coefficient of 10 participant.

**Figure 7:**
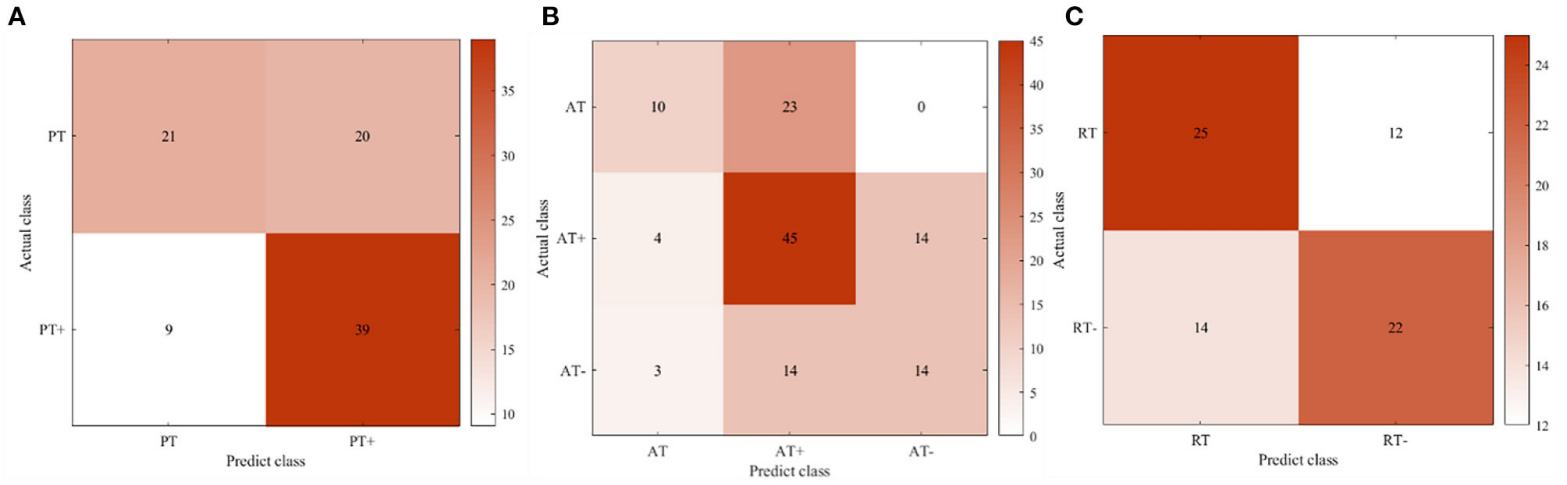
The confusion matrix of participant four in the online experiment. **(A)** Passive training. **(B)** Active training. **(C)** Resistive training.

At the same time, to achieve real-time human-robot interaction, it conducted an online test experiment with the steps which followed the offline experiments. With the assistance of the robot, the participants completed the rehabilitation training experiments of elbow flexion and extension according to seven experimental protocols. The difference was that during the experiment, the offline training classification models would be used to recognize the rehabilitation session of the participants, and the perceived torques estimated by the regression model would be applied to adjust the gain of the control part. The classification accuracy and Kappa coefficient of 10 participants in the three training modes of passive training (binary classification), active training (trinary classification) and resistive training (binary classification) under the online condition are shown in Figure 6, and the confusion matrix of participant 4 is shown in Figure 7.

#### 3.1.2. Results of regression model

**Figure 8:**
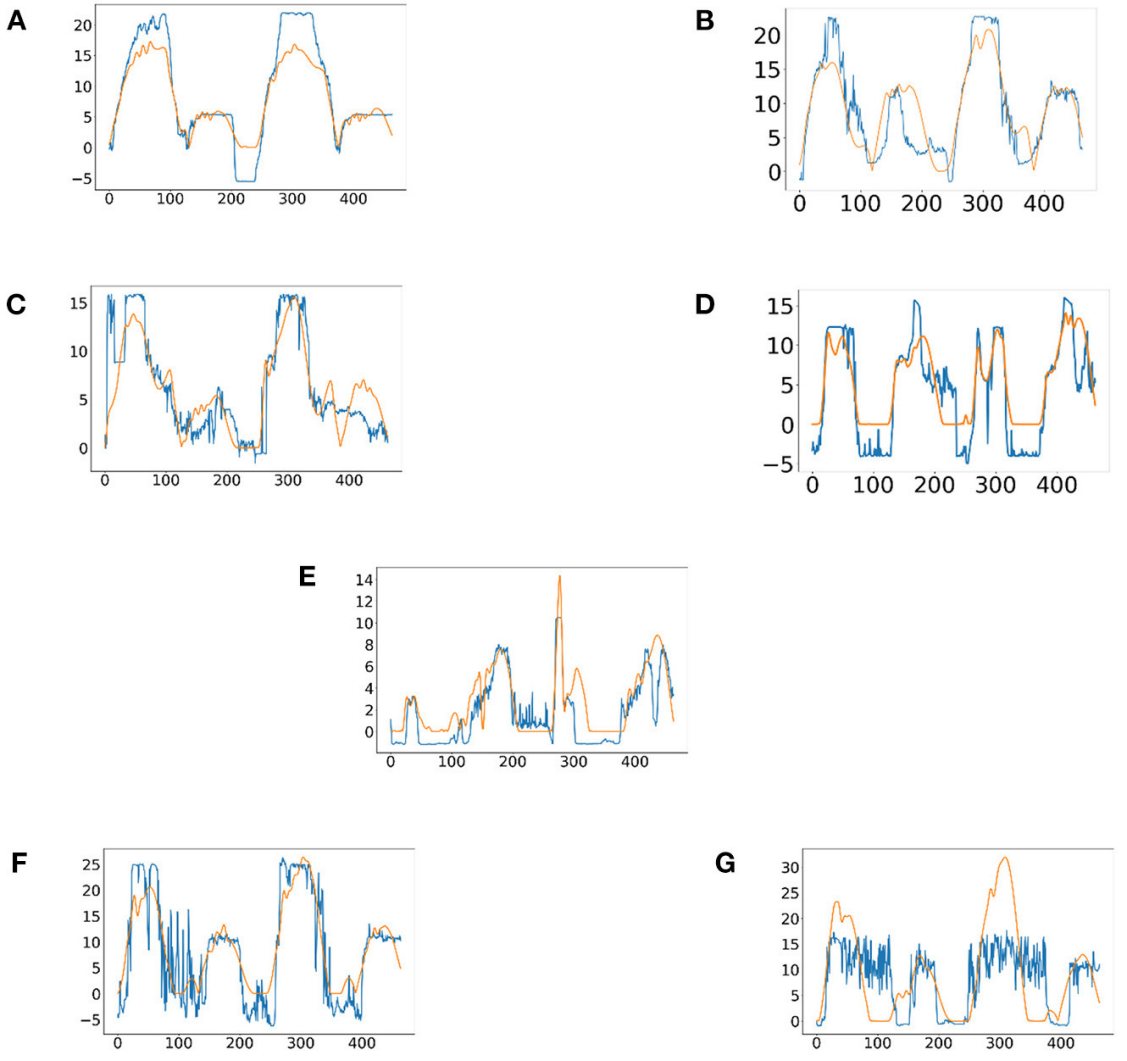
The predicted results of the perceived torques of participant one in seven experimental protocols. **(A)** Passive training with slight motor ability (PT+). **(B)** Complete passive training (PT). **(C)** Active training with weaker motor ability (AT-). **(D)** Normal active training (AT). **(E)** Active training with stronger motor ability (AT+). **(F)** Resistive training with weaker motor ability (RT-). **(G)** resistive training (RT).

In this experiment, it proposed an LSTM-KF model to estimate the perceived torques. Due to the various muscle states in each experimental protocol, the corresponding model fitting ability is also different. Figure 8 shows the predicted perceived torques as blue curves with the referenced values as orange ones. At the same time, among the 7 experimental protocols of 10 participants, the model was evaluated by the test set based on Mean Absolute Error (MAE) loss, as shown in Table 2.

**Table 2:** MAE of 10 participants in seven experimental protocols.

	**PT**	**PT+**	**AT-**	**AT**	**AT+**	**RT-**	**RT**	**Mean**
S1	0.2378	0.1997	0.1706	0.1750	0.1571	0.1850	0.1782	0.1862
S2	0.1564	0.1633	0.1320	0.1576	0.0988	0.2243	0.3530	0.1836
S3	0.2531	0.0144	0.8953	0.7373	0.5829	0.4627	1.6895	0.6622
S4	0.1566	0.1411	0.0980	0.1268	0.2625	0.2113	0.2534	0.1785
S5	0.0973	0.1951	0.1506	0.1540	0.0676	0.2370	0.2644	0.1666
S6	0.1774	0.1805	0.1943	0.1852	0.1667	0.1794	0.2222	0.1865
S7	0.1829	0.1833	0.1703	0.1039	0.1141	0.1934	0.3962	0.1920
S8	0.1675	0.1626	0.1500	0.1640	0.1252	0.2310	0.2390	0.1770
S9	1.7032	0.5814	0.6112	2.2937	3.4626	0.5482	1.0312	1.4616
S10	0.6558	0.2473	0.2733	0.1505	0.2739	1.1969	0.6691	0.4953
MEAN	0.3788	0.2069	0.2846	0.4248	0.5311	0.3669	0.5296	0.3890

### 3.2. Results of AAN control for interaction space reshaping

**Figure 9:**
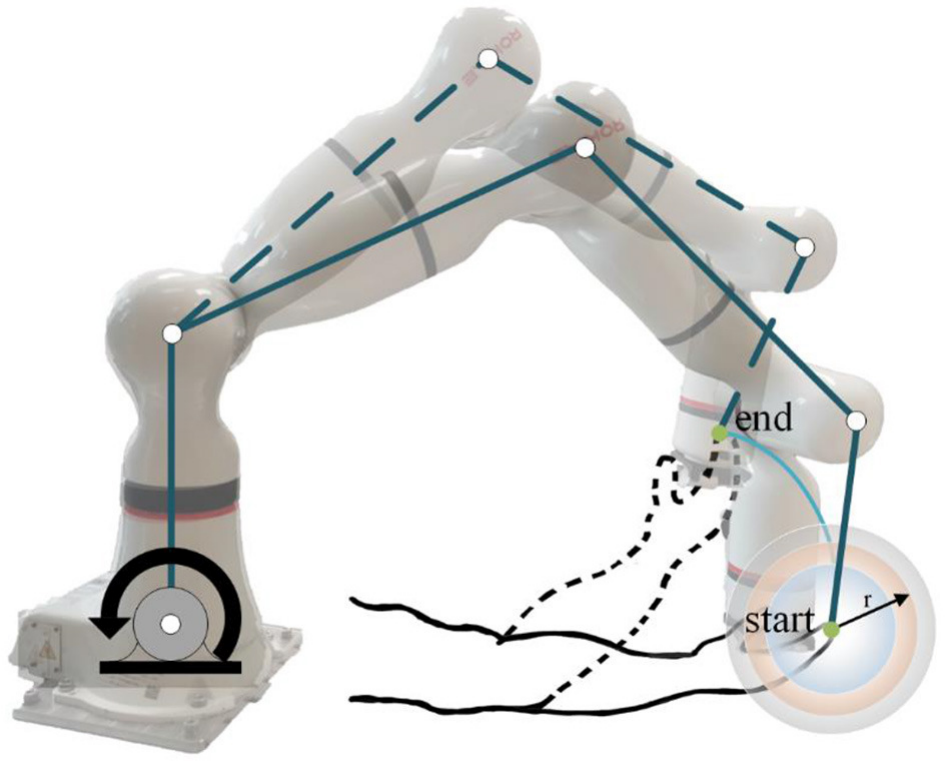
The control outcome of AAN.

The control parameters can be adjusted online in real-time based on the results of the above-mentioned machine learning algorithms to ensure the safety of participants during rehabilitation training and meet the needs of participants in different rehabilitation stages and training sessions. This section mainly carried out online experiments in three different rehabilitation stages and seven protocols to illustrate the effectiveness and safety of the proposed AAN controller for passive, active, and resistive robotic rehabilitation training. Two parameters of the proposed controller were online adjustable to meet the safety requirements in rehabilitation training. These experiments were non-clinical trials to investigate the possible application on patients, and the healthy participants attended to demonstrate that the proposed AAN controller could ensure patients' safety on the desired motion trajectory in rehabilitation training by modifying the control parameters online. The control outcome of AAN in the online experiments and interaction space reshaping is shown in Figure 9.

This paper considers the flexion and extension movement of the upper limbs as the task of the experiment. Therefore, a desired trajectory in Cartesian space is given here as follows


(41) $$\begin{array}{c} \{\begin{array}{c} x=x_{1}-0.15\sin\;\left(wt\right)\\ y=y_{1}\\ z=z_{1}+0.15\sin\;\left(wt\right) \end{array} \end{array}$$

where (*x*_1_, *y*_1_, *z*_1_) are the initial coordinates and they can verify the effectiveness of the proposed controller through the experimental results of participant 1. Set *c*_1_ = 0.1, *c*_2_ = 2.0, *c*_3_ = 5.0, *w* = 0.1*rad*/*s*,*k*_*v*0_ = 10.0,*k*_*p*_ = 6000.0. It could be noted that the aimed trajectory used to verify the proposed AAN control strategy is a two-dimensional trajectory on the XOZ plane. However, the interaction space between the robot and the participants is a three-dimensional spherical space centered around the set trajectory that could be adjusted in real-time. Actually, the participants can freely perform three-dimensional movements in the enveloped spaces of the virtual spheres. Additionally, the AAN control method is not limited to plane or single-joint movements, and the set trajectory can be modified according to specific rehabilitation needs to perform three-dimensional movements or multi-joint movements. The AAN control ensures the safety of patients with the virtual boundary around the aimed trajectory, which also relates to the interaction spaces and assistance levels.

#### 3.2.1. Validation of passive rehabilitation training mode

Passive training is mainly suitable for patients with motor dysfunction in the early rehabilitation stages. In this mode, the rehabilitation robot played a dominant role. Therefore, the participants were required to relax and passively perform repeating training of flexion and extension movement of upper limbs driven by the robot. However, this mode can be subdivided into two protocols, which are protocol-1: complete passive training (PT), and protocol-2: passive training with slight movement ability (PT+). The participants were required to execute these two protocols respectively, therefore the control parameters can be adjusted based on the sEMG signals and kinematic data in real time to develop more detailed rehabilitation training strategies for the patients to ensure the safety of the training sessions by fulfilling the needs of the rehabilitation sessions. Set *r*_*a*_ = 0.04*m, r*_*b*_ = 0.02*m*.

**Figure 10:**
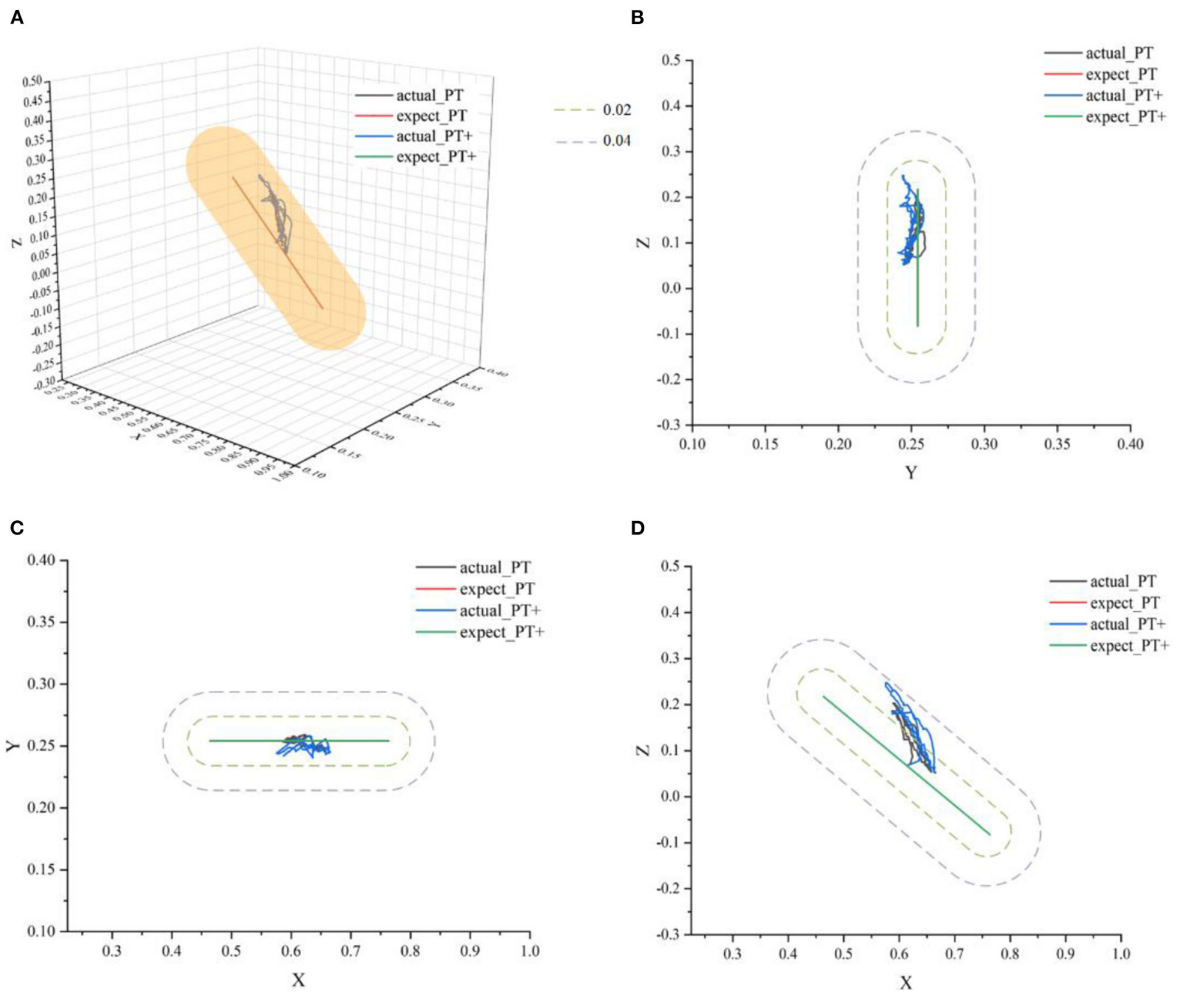
The ideal movement trajectory and the actual training trajectory in the passive training mode: **(A)** three-dimensional spatial view; **(B)** YOZ view; **(C)** XOY view; **(D)** XOZ view.

**Figure 11:**
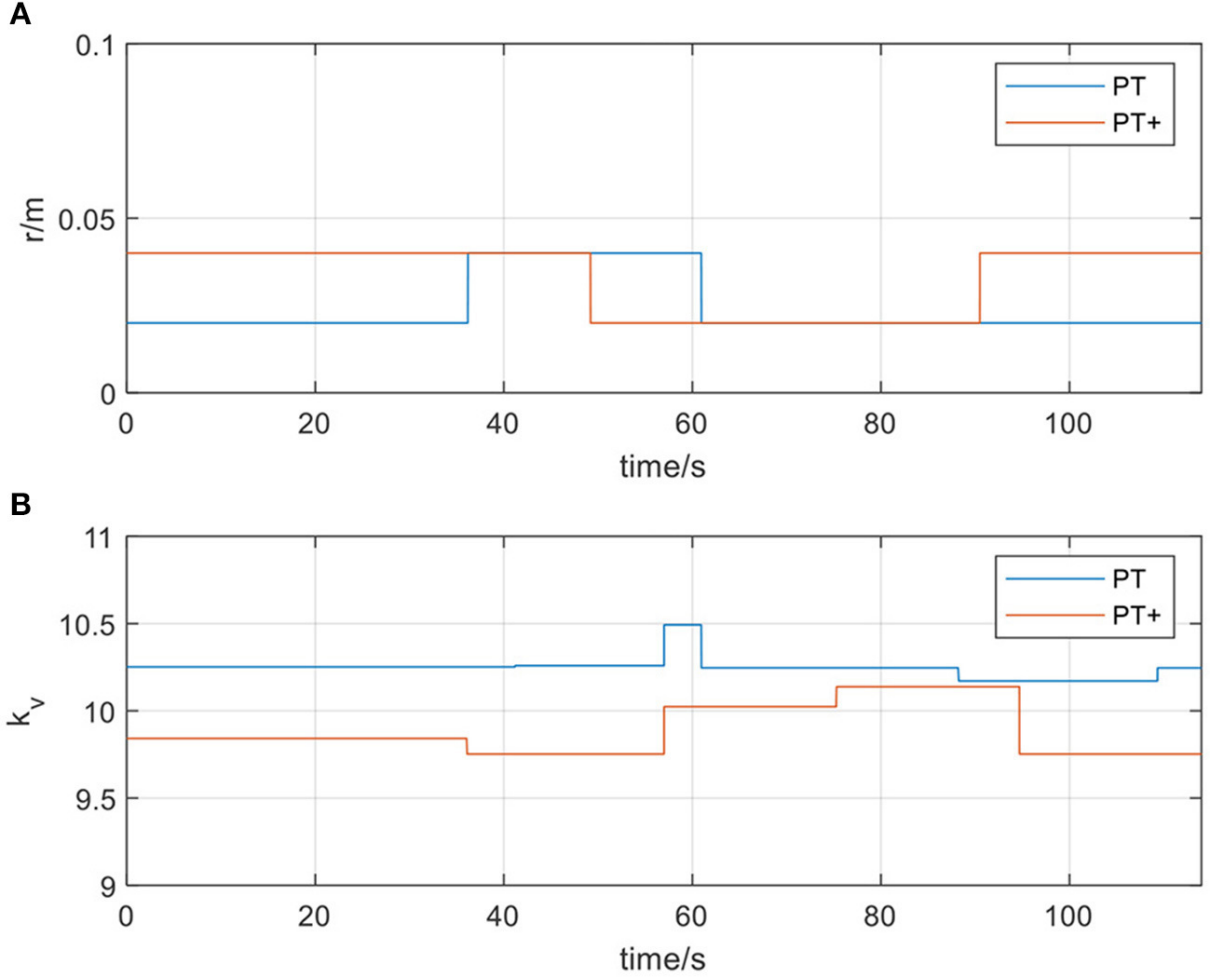
**(A)** The radius of the spherical space in passive training mode; **(B)** The velocity feedback gain *k*_*v*_in passive training mode.

In Figure 10, actual_PT is the training trajectory in the protocol PT, expected_PT is the ideal trajectory in the protocol PT, and actual_PT+ is the training trajectory in the protocol PT+, expected_PT+ is the ideal trajectory in the protocol PT+. Figure 10 shows that in the two protocols in the passive training mode, the actual training trajectories converged in the defined interaction space, thus the experiment results verified the safety and effectiveness of the AAN controller in the passive training mode. From Figure 11A, in the PT and PT+ protocol, the radius of the interaction space can be adjusted online relatively effectively. In the PT protocol, a smaller interaction space was provided for a higher level of safety protection for the participants. A slightly larger interaction space in the PT+ protocol can give participants the interaction space for restoring the normal range of motion of the joints. It can be seen from Figure 11B that the speed feedback gain *k*_*v*_ in the protocol PT is generally larger than that in the protocol PT+ because the participants in this protocol PT simulate a state of flaccid paralysis, and a smaller speed feedback gain can ensure the safety in the training process. The speed feedback gain *k*_*v*_ in the PT+ protocol is larger than that in the PT protocol, which gives the participants relatively wider interaction space in the training to relieve the spasm safely in early rehabilitation training for stroke.

#### 3.2.2. Validation of active rehabilitation training mode

Active training is mainly suitable for the mid-stage rehabilitation of patients with movement disorders after stroke when the patient's motor ability has partially recovered and have certain motor ability during the training. Therefore, in the experiment, participants needed to actively follow the movement direction of the rehabilitation robot to participate in rehabilitation training. In this stage of rehabilitation, three different types of protocols were also considered, namely, protocol-1: normal active training (AT), and protocol-2: active training with weaker motor ability (AT-), and protocol-3: active training with stronger motor ability (AT+). Participants were asked to simulate three different protocols to verify that the AAN controller could ensure the safety of patients in different protocols and adapt to different training needs. Set *r*_*c*_ = 0.10*m, r*_*d*_ = 0.12*m, r*_*e*_ = 0.14*m*.

**Figure 12:**
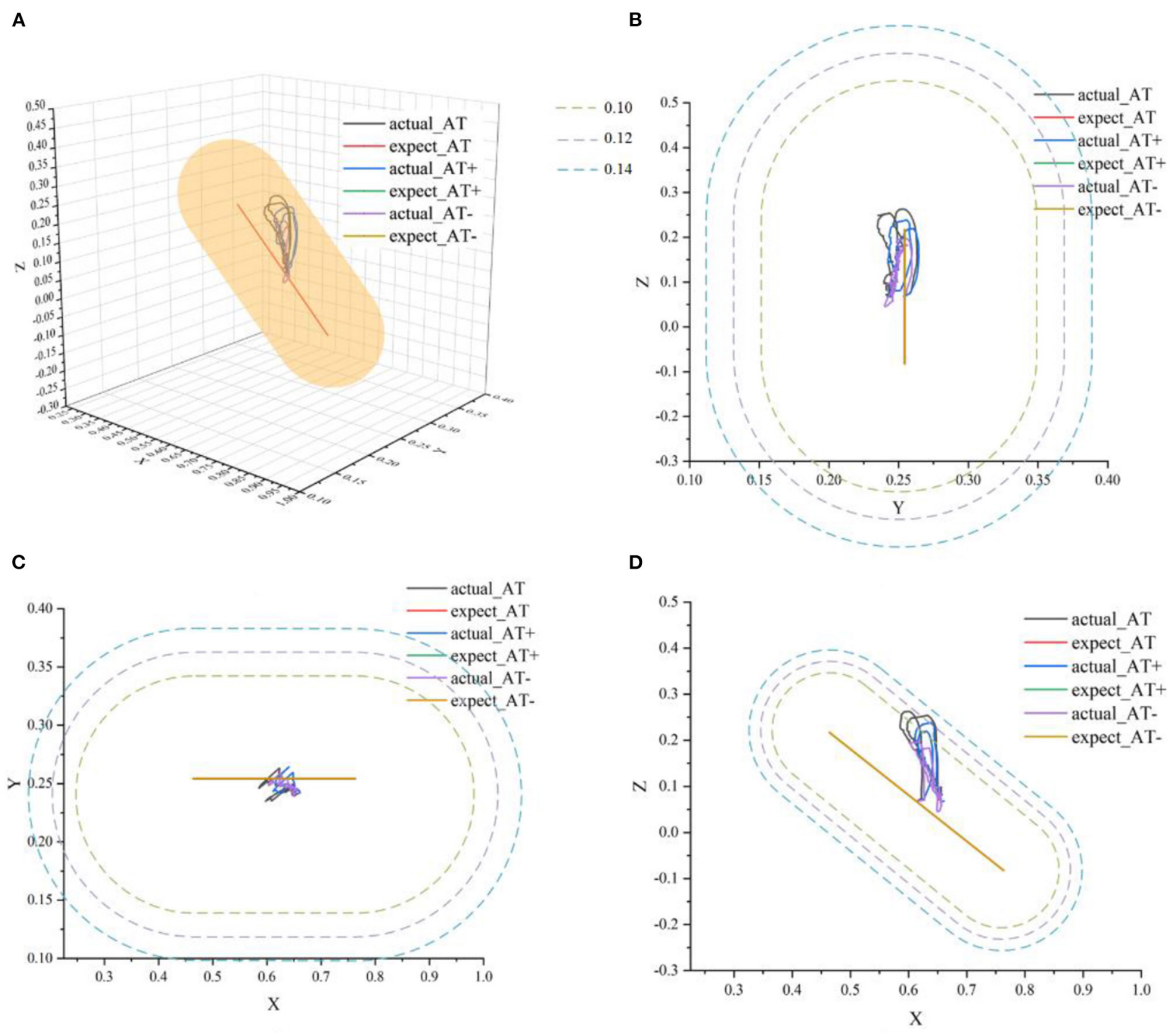
The ideal movement trajectory and the actual training trajectory in the active training mode: **(A)** three-dimensional spatial view; **(B)** YOZ view; **(C)** XOY view; **(D)** XOZ view.

**Figure 13:**
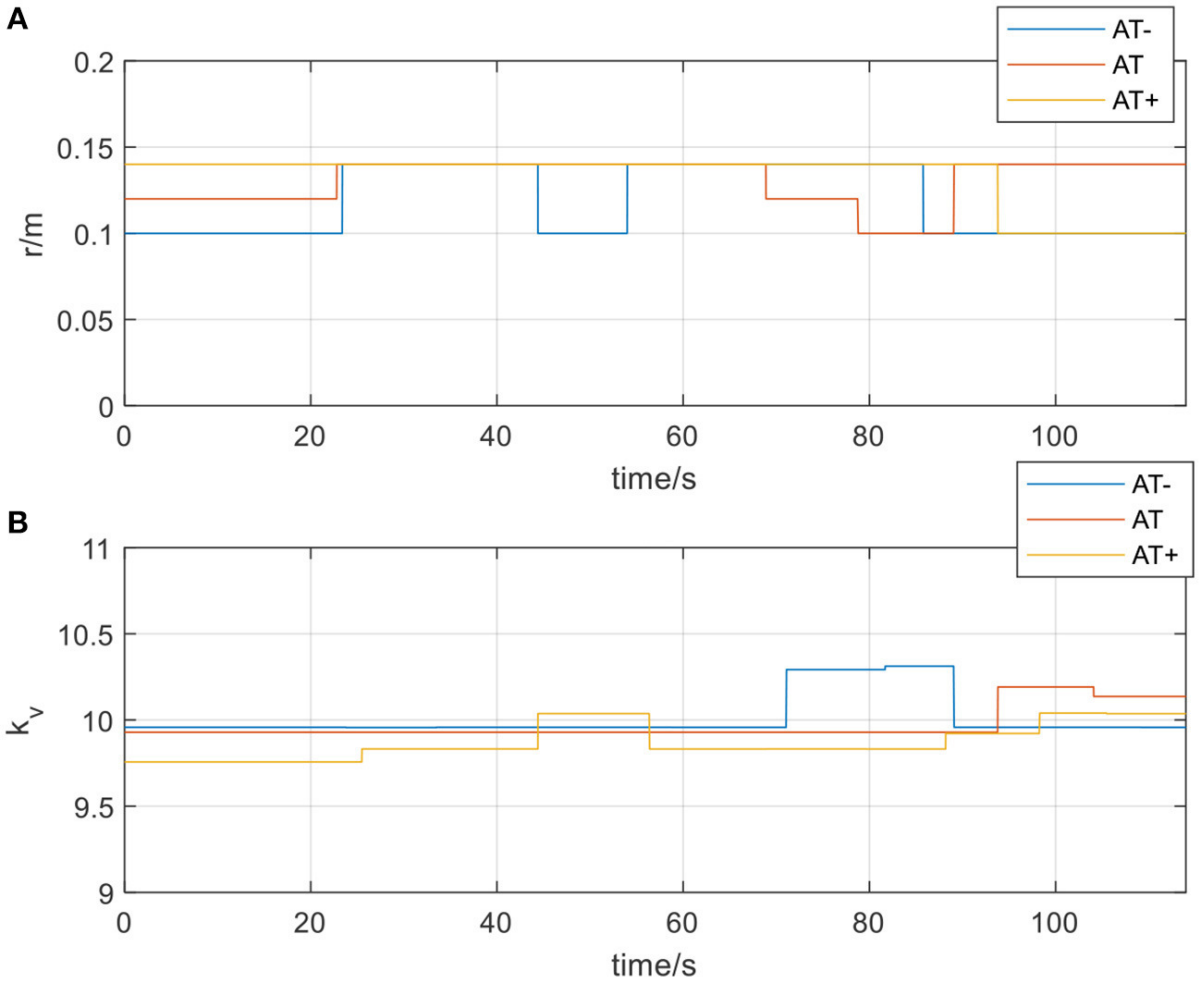
**(A)** The radius of the spherical space in active training mode; **(B)** The velocity feedback gain *k*_*v*_in active training mode.

In Figure 12, actual_AT is the training trajectory in the protocol AT, expected_AT is the ideal trajectory in the protocol AT, actual_AT+ is the training trajectory in the protocol AT+, expected_AT+ is the ideal trajectory in the protocol AT+, actual_AT- is the training trajectory in the protocol AT-, expected_AT- is the ideal trajectory in the protocol AT-. Figure 12 shows that the training trajectories in the three protocols in the active training mode are all restricted to the interaction space defined separately, which verifies that the proposed AAN controller can ensure the training safety of the participants in the three protocols in the active training mode. Figure 13A shows that in the online experiments, the radius of the interaction space of three protocols satisfies: r (AT-)< r (AT)< r (AT+). It is because, with the gradual increase of muscle strength, patients need to be given larger interaction space to improve the quantity of training by enhancing the engagement with safety interaction. Figure 13B shows that in the active training, the patient has recovered with certation motor abilities and needs to obtain sufficient assistance amount in the training sessions. Therefore, the speed feedback gain *k*_*v*_ in the online experiment in the three protocols decreases in turn to match the gradually increased muscle strength of the patients for activating their rehabilitation intentions.

#### 3.2.3. Validation of resistive rehabilitation training mode

Patients in the later stages of stroke rehabilitation have basically recovered their motor ability, but their upper limb muscle strength is still insufficient, so they need to intensify their muscle strength through resistive training. In the experiment, the participants were asked to perform the training in the opposite direction to the movement direction of the rehabilitation robot. Given the physical demands of resistance training, sudden loss of strength and drop is likely to occur during training. Therefore, in the resistive training mode, it can also be subdivided into two types of protocols, protocol-1: resistive training (RT) and protocol-2: resistive training with weaker movement ability (RT-). Similarly, the participants were required to simulate two protocols in the resistive training to verify the correctness and safety of the AAN controller. Set *r*_*f*_ = 0.06*m, r*_*g*_ = 0.08*m*.

**Figure 14:**
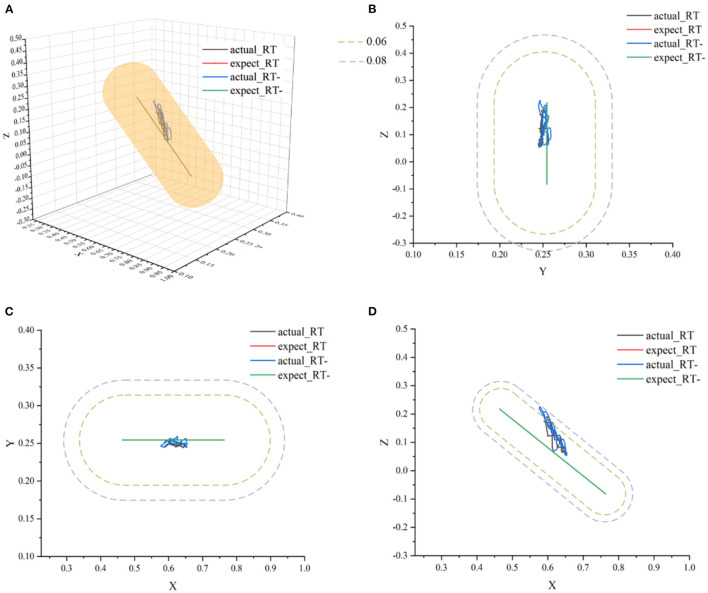
The ideal movement trajectory and the actual training trajectory in the resistive training mode: **(A)** three-dimensional spatial view; **(B)** YOZ view; **(C)** XOY view; **(D)** XOZ view.

**Figure 15:**
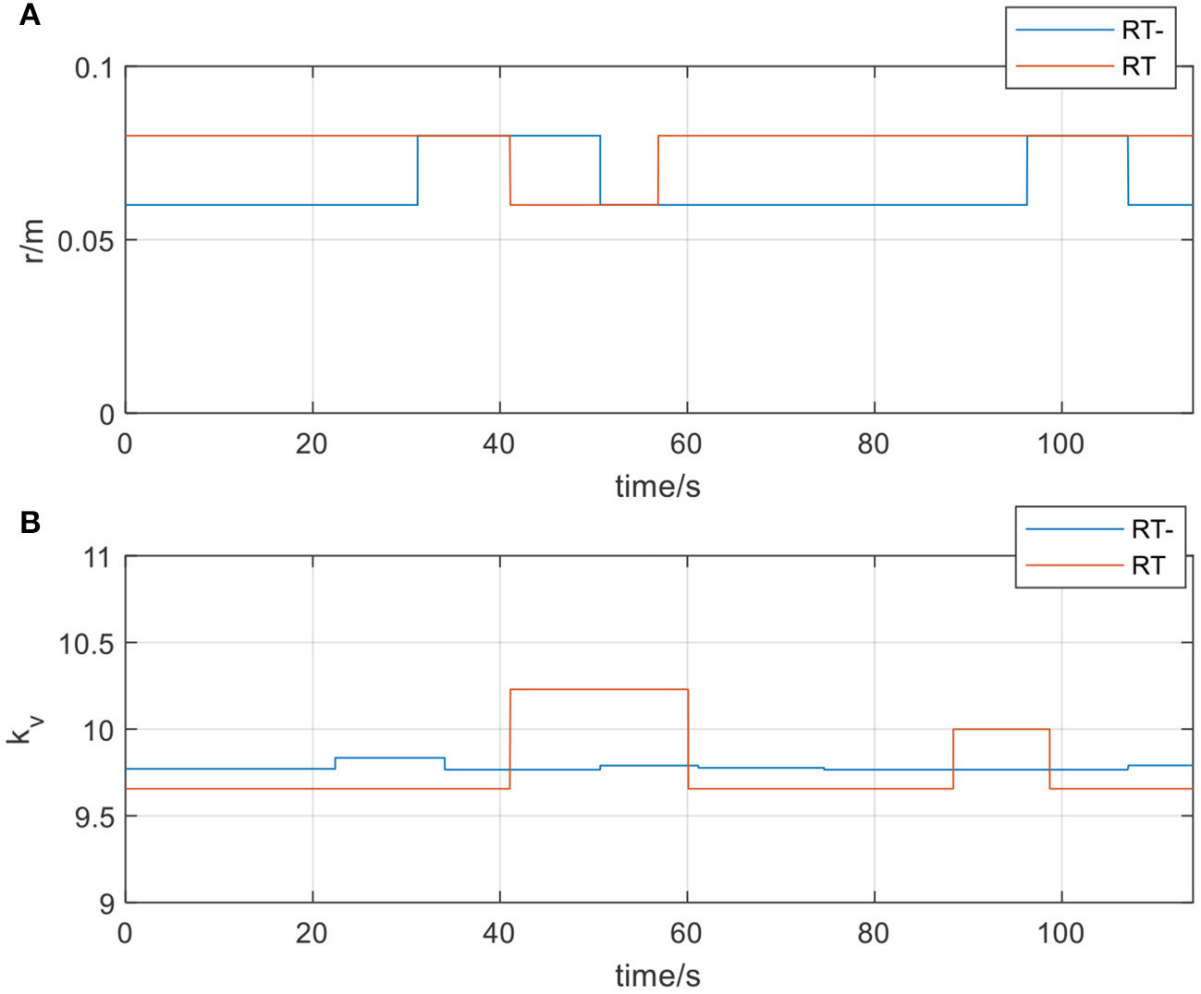
**(A)** The radius of the spherical space in resistive training mode; **(B)** The velocity feedback gain *k*_*v*_ in resistive training mode.

In Figure 14, actual_RT is the training trajectory in the protocol RT, expected_RT is the ideal trajectory in the protocol RT, and actual_RT- is the training trajectory in the protocol RT-, expected_RT- is the ideal trajectory in the protocol RT-. As shown in Figure 14, the actual training trajectories in the online experiments of two protocols in the resistive training mode converged within the region of the defined interaction space respectively. Figure 15A presents the interactive space adjustment results of online experiments in the resistive training mode, and relatively stable adjustment can be achieved in two protocols. The RT- protocol is designed considering the sudden decrease of strength in resistive training, so compared with the RT protocol, it needs a relatively smaller interaction space to ensure the safety of the participants, and the experimental results verified the effectiveness of this protocol design. The regulation of velocity feedback gain *k*_*v*_ in Figure 15B also reflects the different requirements between the two protocols in resistive training mode. The value of *k*_*v*_ in RT- protocol is generally smaller than that in RT protocol because RT protocol requires a larger velocity feedback gain to ensure the safety of the participant in the case of a sudden decrease of force or muscle fatigue.

### 3.3. The quantification of the assistance in AAN control

After the stroke, kinematic measures obtained with robotic and non-robotic devices are highly recommended to precisely quantify the sensorimotor impairments of the upper limbs which influence the selection of therapeutic strategies (Treussart et al., 2020). In the upper limb rehabilitation training, the engagement of patients in the AAN control closely relates to the assistance level of the robot, which could be discussed from the perspective of human-robot interaction. In the interaction, the assistance level determines the amount of support provided by the assistant (Passenberg et al., 2011), and this level of rehabilitation training should be described quantitatively. Although the robots themselves are described as suitable to record movement data such as position, velocity and joint torques, and the quantitative reliable measurement of kinematics and dynamics is implemented during recovery by these parameters (Nataliya et al., 2017), while the measurement system has the impact on the performed movements due to the inertia and arm weight of the robot (Schwarz et al., 2019). In this paper, the speed feedback gain *K*_*v*_ based on the perceived torques is applied to fulfill the engagement needs, and it should be further discussed whether the perceived torques relate to the quantified assistance levels. Because it is a cooperative process when the patient is assisted by the robot during the rehabilitation, the assistance level could be measured by the accumulated motion perception in a training session rather than the single torque. In this paper, the perceived torques were estimated by the proposed LSTM-KF method. The sum of the torques of the patient in the rehabilitation session was measured by the summation. The sum of the torques of the patient was defined as TPT (Total Perceived Torques) and expressed as the formula:


(42) $$\begin{array}{c} TPT=\overset{t_{T}}{\underset{t=0}{\sum }}\|T_{p}\| \end{array}$$

Here, *t*_*T*_ is the current time corresponding to the number of discrete upper limb joint rotation steps, *T*_*p*_ is the perceived torque, and ‖·‖ is the two-norm. With the estimation of the perceived torque in discrete time steps, the TPTs from the 8 participants are calculated in Table 3.

**Table 3:** Total perceived torques values of 10 participants in seven experimental protocols.

	**PT**	**PT+**	**AT-**	**AT**	**AT+**	**RT-**	**RT**
S1	5400.55	4555.31	3159.53	3135.66	2733.77	4386.09	5152.81
S2	4630.22	4311.25	2886.18	3093.00	1176.41	4813.02	3905.10
S3	4088.47	4265.15	2376.27	2895.42	2702.52	5469.25	5942.42
S4	3826.01	3409.05	3201.36	1762.58	4982.95	5036.48	5907.66
S5	3734.77	4127.71	2587.66	2698.64	816.17	3546.80	4762.83
S6	3920.90	4446.01	3706.18	2683.19	2548.39	4237.62	4719.10
S7	3816.12	5903.69	2521.68	1718.20	1739.73	5019.03	5177.91
S8	4205.00	3929.18	3233.89	3640.02	2608.22	3283.41	4060.02
S9	3644.98	5335.62	4403.53	4208.14	3428.40	3418.65	3897.73
S10	5358.99	5041.23	3573.60	3504.55	4080.19	4034.72	4623.92

From Table 3, it can be seen that the TPT value of active training in the three protocols is the smallest, and that in resistive training is the largest for most participants.

Considering the difference between participants, the normalized mean of perceived torque is further calculated as formula (43).


(43) $$\begin{array}{c} NMPT=\frac{\frac{1}{N}\sum_{t=0}^{s_{T}}\|T_{p}\|}{\underset{P}{\max}\;\left(\frac{1}{N}\sum_{t=0}^{t_{T}}\|T_{p}\|\right)} \end{array}$$

Here, $\frac{1}{N}\sum_{t=0}^{s_{T}}\|T_{p}\|$ is the mean value of the interaction torque in different protocols, and $\underset{PT}{\max}\frac{1}{N}\sum_{t=0}^{t_{T}}\|T_{p}\|$ is the maximum mean value in the PT training. With TPT from the 10 participants, the NMPT is obtained in Table 4.

**Table 4:** Normalized mean perceived torque value of 10 participants in seven experimental protocols.

	**PT**	**PT+**	**AT-**	**AT**	**AT+**	**RT-**	**RT**
S1	1.00	0.84	0.59	0.58	0.51	0.81	0.95
S2	1.00	0.93	0.62	0.67	0.25	1.04	0.84
S3	0.96	1.00	0.55	0.69	0.64	1.30	1.41
S4	1.00	0.90	0.84	0.46	1.31	1.36	1.55
S5	0.92	1.00	0.64	0.66	0.20	0.88	1.14
S6	0.87	1.00	0.83	0.61	0.59	0.91	1.02
S7	1.00	0.99	0.64	0.43	0.44	1.27	1.32
S8	1.00	0.93	0.78	0.87	0.32	0.78	0.97
S9	0.70	1.00	0.83	0.83	0.64	0.64	0.74
S10	1.00	0.91	0.68	0.66	0.75	0.76	0.87

**Figure 16:**
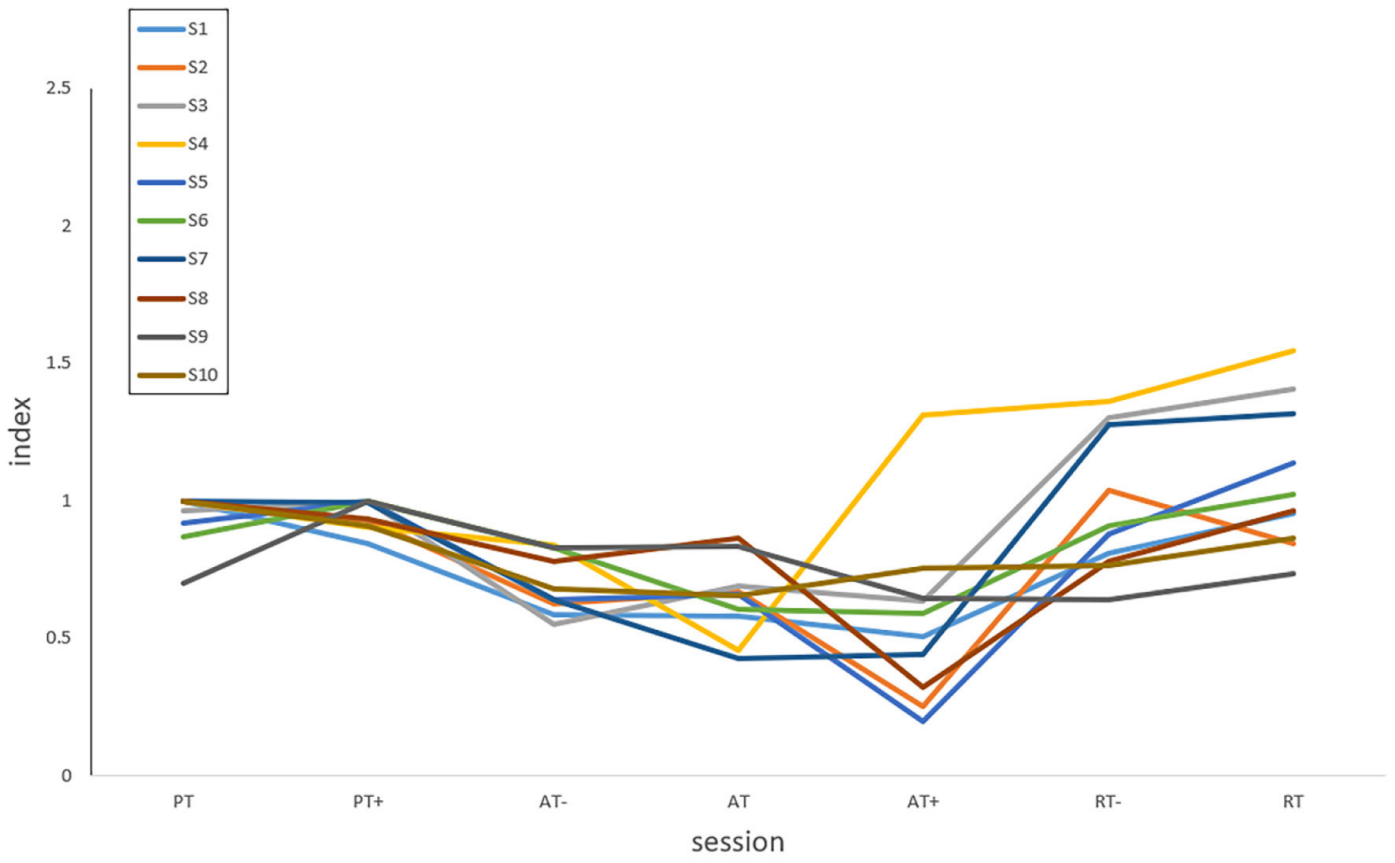
The index of assistance level of 10 participants in seven experimental protocols.

The results of NMPT could be analyzed according to the rehabilitation training stages and sessions. For the elbow flexion and extension movements in passive and active training, the upper limb rotation direction of the participant is consistent with that of the robot. The greater the muscle strength of the participant, the smaller the perceived torques and the value of NMPT, which represents better engagement and motor ability. In the resistive training, the larger NMPT the better because the motion intention of the patients has the opposite directions to the robot, and thus the engagement and motor ability can be still expressed by NMPT. The NMPT could be interpreted as the quantified assistance level in the AAN control and reveal the engagement of patients, especially in the subdivided rehabilitation stages and sessions for the unstable states of the patients during their stroke recovery processes. On this basis, the index of assistance level of the participant is defined from the NMPT values in all training sessions. To visualize the index of assistance level, the NMPT values of 10 participants in seven experimental protocols data are plotted in Figure 16.

## 4. Discussion

Current work focuses on the variations of patients' motor ability in rehabilitation training stages and sessions and the way to map the changes in the motor ability to these stages and sessions. It is expected to recognize the current rehabilitation training stages and sessions of the patients, and then adjust the workspace of the robot according to these stages and sessions for safe AAN control. In the offline experiments, participants were required to carry out experiments in the order of PT, PT+, AT-, AT, AT+, RT-, RT, while in the online experiments, the switch of the rehabilitation sessions of participants was uncertain and changed according to the intention of participants randomly. In the offline phase, one or two participants were enrolled to conduct the experiment each day. With a sufficient amount of data, the data set was divided into a training set and a test set in a ratio of 9:1, and the classification model and regression model were trained. A total of 10 participants' offline experimental data were collected, and the same 10 participants attended the online experiment on another day. The situation is different at the beginning of the actual applications because the data of specific patients is difficult to access. Considering this problem and the difference among individuals, the patients followed the movement of the robot at the beginning of the training, and a small amount of EMG data was collected because SVM is suitable for small sample learning (Khairuddin et al., 2021). With the progress of the rehabilitation experiment, it would acquire more data on patients at various stages and sessions of rehabilitation, which could be utilized as the input for further algorithm model training for specific patients.

In offline experiments, it could be seen that the average accuracy of the classification model is mostly above 95% in Table 1. An upper limb motion pattern recognition method for the rehabilitation robot ReRobot is proposed using sEMG signals and SVM, and the classification accuracy is 94.18% (Cai et al., 2019), which indicates that the classification of surface electromyography data for rehabilitation training in this paper is feasible. they distinguish patients' motor ability in each stage, to provide corresponding control strategies for the rehabilitation robot according to the progress of rehabilitation, which serves for purpose of AAN in human-robot interaction. In online experiments, Figure 6 shows that the accuracy of the three training stages reaches 73.47, 61.61, and 68.07%, and Figure 7 shows that the highest Kappa coefficient is 0.4740, 0.3701, and 0.3553. These multi-class classification results have better accuracy than 55–70% for online applications (Zhang et al., 2018), demonstrating that the proposed rehabilitation system can distinguish training sections in different rehabilitation stages in real time and adjust the rehabilitation robot based on the motor ability of the patients.

From the results of the regression model shown in Figure 8, it could be seen that the regression model was particularly superior in the complete passive training (PT) and passive training with slight motor ability (PT+) paradigm, with remarkable accuracy and tracking performance. This was because the motor ability of the participants under the protocol was relatively stable and predictable. By contrast, the performance of the model in the resistive training (RT) protocol was slightly inadequate, the sudden variation trend sometimes had not been well predicted, and a certain delay could also be observed, which was because in this protocol, the muscle strength of the participant might suddenly decrease due to continuous loading leading the emergent drop process, and the sudden variation appears on the patient's sEMG data and noises became obvious. The drop often occurs in a short time, thus it has comparatively low prediction accuracy. Meanwhile, to verify the accuracy of the regression model, MAE results were discussed next. In Table 2, except for participant three and participant nine, MAE values of the seven experimental protocols of the other participants are all <0.5, indicating that the proposed regression model had a low prediction error and could have sufficient performance in the estimation of the interaction torque (Kingma and Ba, 2014). Meanwhile, through longitudinal comparison, it could be found that PT, AT and RT- have the lowest MAE values, reflecting that the regression model of these three experimental protocols has a better fitting ability. On the whole, the total mean of MAE was 0.3890, which proved that the LSTM-KF method had better performance to estimate the perceived torque of participants in real-time, which provided the possibility to adjust the control parameter online and quantify the assistance level for further clinical application. Additionally, prediction models that generalize to new subjects would be helpful (Gerig et al., 2017). For the condition without the sEMG data of the new patient beforehand, further cross validation was attempted, which utilized nine participants to train the classification model and left one out each time to verify the classification model performance. This method was used to cross-verify all 10 participants' data in three training stages, and the classification accuracy was obtained in Table 5. It could be seen that most classification accuracy values were lower than the trained individual models for each participant, and all the average classification accuracy values of trained general models in the three training stages were less than that of the trained individual models. However, it is an alternative approach at the beginning of the experiment by using the general model to classify the data of the new participants. As the rehabilitation training continues, enough sEMG data of participants in various rehabilitation stages and sessions are obtained, which could be utilized as the input of the individual classification model training for specific patients, and then the accuracy and efficiency of the model would be improved.

**Table 5:** The classification accuracy of different models for 10 participants in three training stages.

	**Passive**		**Active**		**Resistive**	
	**Individual model**	**General model**	**Individual model**	**General model**	**Individual model**	**General model**
S1	73.47%	57.33%	52.50%	40.36%	60.94%	57.03%
S2	59.73%	62.69%	57.81%	46.91%	62.89%	73.39%
S3	58.50%	50.45%	52.70%	39.26%	54.90%	56.86%
S4	67.42%	50.68%	54.33%	49.61%	64.38%	65.75%
S5	64.20%	61.73%	61.59%	52.52%	59.78%	57.14%
S6	63.81%	64.76%	61.61%	45.11%	67.44%	52.81%
S7	65.81%	64.10%	51.35%	58.61%	54.64%	53.28%
S8	55.10%	59.63%	58.27%	58.48%	66.30%	64.65%
S9	61.63%	56.98%	57.89%	50.32%	56.44%	59.41%
S10	69.77%	53.49%	52.35%	44.62%	68.07%	56.25%
Average	63.94%	58.18%	56.04%	48.58%	61.58%	59.66%

The index of assistance level reflects the motor ability and rehabilitation sessions of patients. In order to prove whether the rehabilitation training in this experiment is reasonable, the index of assistance level was analyzed. From Figure 16, it can be seen obviously that the assistance level of active training in the three protocols was relatively smaller, but larger in the passive training for most participants, and those of PT sessions were close to PT+ sessions with an index around 1.0, which represents the variation of the needs of patients with a baseline by training sessions. In passive and active training, the upper limb completed the rotation consistent with the movement of the robot. The smaller the index of assistance level, the better the participation and motor ability. In resistive training, the participants should attempt to resist the robot with opposite motion intention following the rotation. As a result, it becomes a type of assistance to strengthen motor ability and does not necessarily have a higher level than that of passive and active training, which is usually used in the late stage of stroke recovery. Unlike passive and active training, the index of assistance level increases with the improvement of the motor ability in resistive training. To prevent the sudden drop of the upper limb to the opposite direction, the assistive torque value from the robot was set in a relatively safe range which caused some index of assistance level smaller than 1.0. In actual rehabilitation training, the motor ability would not always change from PT- to RT by sessions as Figure 16, but the index values are still capable of reflecting the variation of the assistance needs from patients in training sessions belonging to the passive, active or resistive training stage.

It also could be found that most of the two sessions of passive training had little difference in the overall trend because the patients had unstable states during the early rehabilitation stages without enough muscle strength, and in the three sessions of the active training stage, decreasing assistance level was presented as the strength of the patient increases in the recovery process. Participant S4 is a special case in the active training stage. The exceptional index value of assistance level in AT+ was caused by muscle fatigue in AT. Due to the decline of the strength, the motor ability could not reach the expected range of AT+ which was even lower than that of AT. In the two resistive sessions, the RT session was larger than that of the RT- session, which represented the consolidation of the rehabilitation outcomes. The quantification of assistance level characterizes the engagement needs of patients by training stages and sessions for reshaping human-robot interaction spaces safely, facilitating the clinical application of the upper limb rehabilitation robot. If the results are provided to the therapist for motor ability analysis with medical implications (Murphy and Hger, 2015), the detailed range of the index in each recovery state should be studied with a larger number of participants in different stroke recovery stages.

## 5. Conclusion

It focuses on upper limb rehabilitation facing all the passive, active and reactive training stages for stroke patients. With the designed experimental protocols, the training sessions are graded from the perspective of the progress of rehabilitation, and the sEMG data are collected according to the subdivided motor ability. To achieve AAN control, the output of the PSO-SVM classifier reshapes the interaction space with the spherical virtual boundary, and the LSTM-KF model predicts the perceived torques to provide the gain modification. In this way, the safety of the rehabilitation is ensured by the region controller in each training session. From the perspective of human-robot interaction, the quantified assistance level characterizes the needs of the patients in AAN control by analyzing the results of 10 groups of experiments.

In the current study, only two sEMG electrodes are attached to the upper limb of the participant. The sEMG data classification results work as the input of AAN control, which mainly aims at verifying the feasibility of the human-robot interaction space reshaping by recognizing recovery states. In future work, more sEMG electrodes will be placed on the upper limb, and the classification algorithm performance could be promoted with more complete muscle activation data. Additionally, stroke patients in different recovery stages rather than healthy participants will attend the training based on the proposed interaction space reshaping method, and the real-time performance of the system would be evaluated. By sufficiently considering the engagement of the patients, it could have more potential to apply in clinical upper limb rehabilitation training with the quantified assistance level.

## Data availability statement

The raw data supporting the conclusions of this article will be made available by the authors, without undue reservation.

## Ethics statement

The studies involving human participants were reviewed and approved by the Ethics Committee of West China Hospital of Sichuan University. The patients/participants provided their written informed consent to participate in this study.

## Author contributions

XL and PC wrote the manuscript. HH analyzed of the clinical problems. QL and SG implemented the AAN control and machine learning methods. XY provided the suggestion of experiment protocols design. PC and KL worked on the assistance level quantification. All authors contributed to the article and approved the submitted version.
